# Directed evolution of orthogonal RNA–RBP pairs through library-vs-library *in vitro* selection

**DOI:** 10.1093/nar/gkab527

**Published:** 2021-07-05

**Authors:** Keisuke Fukunaga, Yohei Yokobayashi

**Affiliations:** Nucleic Acid Chemistry and Engineering Unit, Okinawa Institute of Science and Technology Graduate University, Onna, Okinawa 904 0495, Japan; Nucleic Acid Chemistry and Engineering Unit, Okinawa Institute of Science and Technology Graduate University, Onna, Okinawa 904 0495, Japan

## Abstract

RNA-binding proteins (RBPs) and their RNA ligands play many critical roles in gene regulation and RNA processing in cells. They are also useful for various applications in cell biology and synthetic biology. However, re-engineering novel and orthogonal RNA–RBP pairs from natural components remains challenging while such synthetic RNA–RBP pairs could significantly expand the RNA–RBP toolbox for various applications. Here, we report a novel library-vs-library in vitro selection strategy based on Phage Display coupled with Systematic Evolution of Ligands by EXponential enrichment (PD-SELEX). Starting with pools of 1.1 × 10^12^ unique RNA sequences and 4.0 × 10^8^ unique phage-displayed L7Ae-scaffold (LS) proteins, we selected RNA–RBP complexes through a two-step affinity purification process. After six rounds of library-vs-library selection, the selected RNAs and LS proteins were analyzed by next-generation sequencing (NGS). Further deconvolution of the enriched RNA and LS protein sequences revealed two synthetic and orthogonal RNA–RBP pairs that exhibit picomolar affinity and >4000-fold selectivity.

## Graphical Abstract

**Graphical Abstract:**
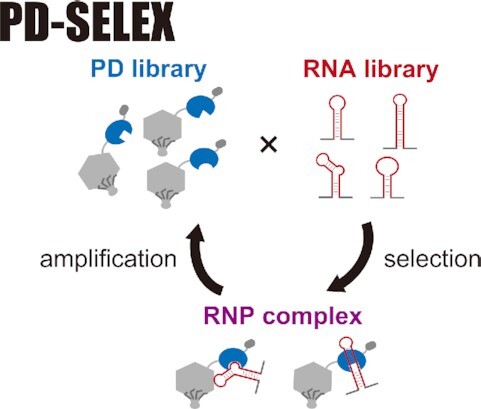
Library-vs-library selection of RNA and RNA-binding protein (RBP) resulted in orthogonal RNA–RBP pairs with picomolar affinity and high selectivity.

## INTRODUCTION

RNA and RNA-binding protein (RBP) pairs, natural or engineered, are used as adaptors to link an RNA of interest with a protein of interest for various applications. For example, the specific interaction between the bacteriophage MS2 coat protein (MS2CP) and MS2 RNA stem–loop (1) has been exploited for fluorescence imaging of cellular mRNAs (2,3), proximity biotinylation of proteins using the labeling enzyme APEX2 (4), isolation of small non-coding RNA-binding proteins (5), transcriptional activation with engineered CRISPR–Cas9 complex (6–8), and post-transcriptional gene circuits (9–11). However, the number of RNA–RBP pairs used in these emerging applications, especially for mammalian synthetic biology, is limited (12).

A reasonable strategy to generate novel RNA–RBP pairs is to re-engineer the binding characteristic of an existing RNA–RBP pair. This has typically been done in two steps. (i) Mutate either the parental RNA or RBP to abolish binding (13,14). (ii) Construct a wholly or partially randomized library of the binding partner (RNA or RBP) and perform affinity selection (13,15). The first step is essentially designing a loss-of-function mutant which is relatively straightforward if the structural information (e.g. crystal structure) is available. However, such a variant may not be optimal for binding a novel partner. The second step is a canonical affinity selection based on established methods such as Systematic Evolution of Ligands by EXponential enrichment (SELEX) (16,17) and Phage Display (PD) (18–20).

While these in vitro selection technologies usually involve the selection of an RNA or protein library for binding to a single target, an alternative approach is library-vs-library selection (or screening) which has been employed for identification of protein–protein (21–25), protein–peptide (26), peptide–peptide (27,28), small molecule–protein (29,30) and small molecule–RNA interactions (31). To our knowledge, however, RNA library-vs-protein library selection has not yet been reported.

**Figure 1.**
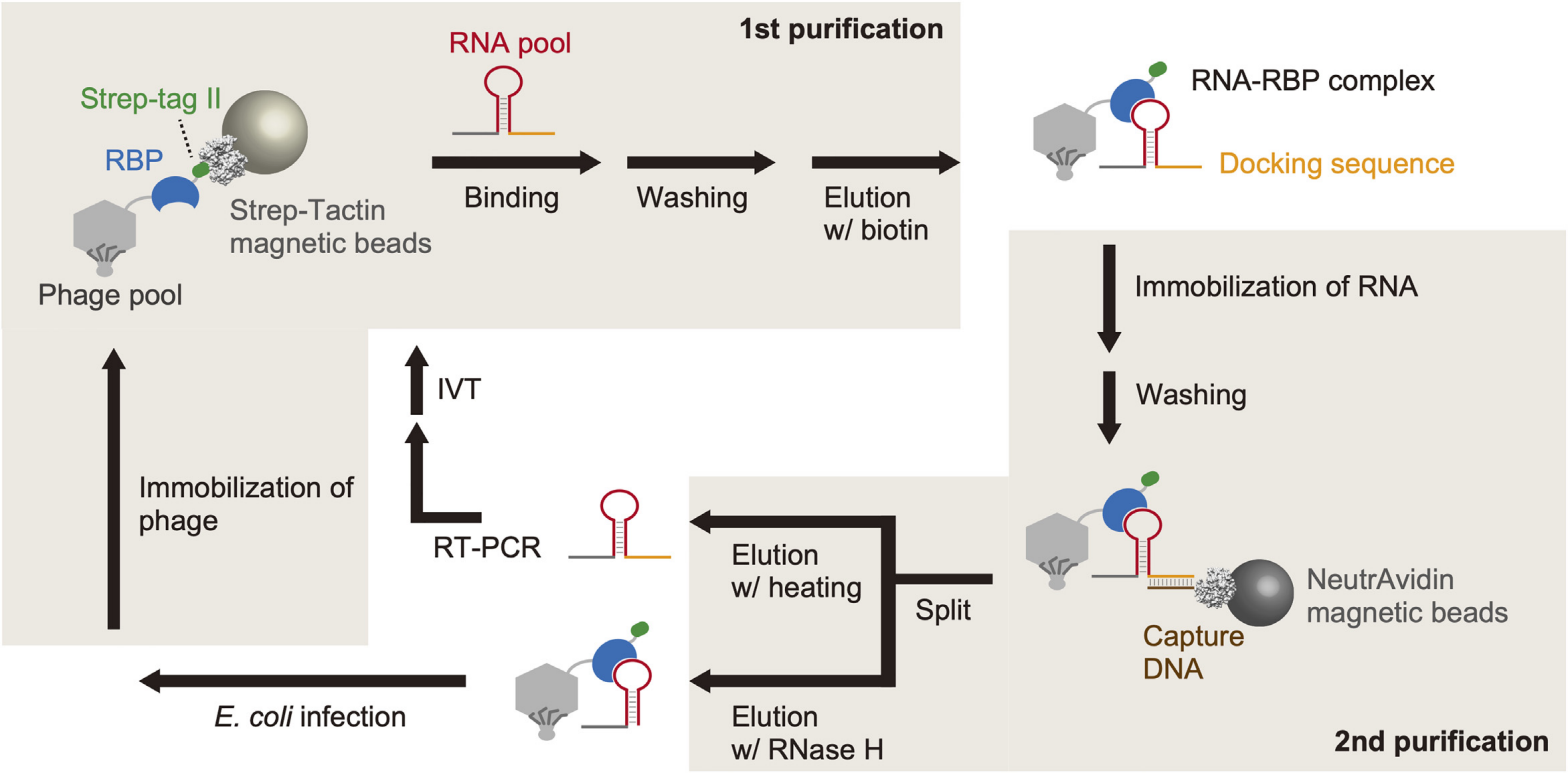
Schematic illustration of PD-SELEX. RBPs are displayed on T7 phage and immobilized on Strep-Tactin magnetic beads via Strep-tag II fused to the C-terminus of RBP. After capturing RBP-binding RNAs, the phage particles are eluted, and phage-displayed RBP–RNA complexes are captured by magnetic beads immobilized with an oligo DNA complementary to the fixed RNA sequence (docking sequence). RNA and phage particles are separately eluted and amplified for the subsequent round of selection.

Here, we combined PD with SELEX (PD-SELEX) to develop a method to coevolve RNAs and RBPs through library-vs-library in vitro selection (Figure 1). First, we optimized the selection protocol using the box C/D kink-turn (Kt) RNA and L7Ae (32) as a model RNA–RBP pair. This was partly motivated by the frequent use of L7Ae and its RNA target motifs in synthetic biology applications, for example, mRNA delivery (33), mRNA loading into exosomes (34), and gene switches in vitro (32,35), in *Escherichia coli* (36), and in mammalian cells (9,11,12,32,37–45). We then constructed a PD library displaying L7Ae protein mutants randomly mutated at 8 surface residues and a partially structured RNA library to execute library-vs-library selection (PD-SELEX). The enriched RNA–RBP pairs were deconvoluted by conventional single-RBP versus RNA library selections, and the binding affinity and selectivity of the discovered RNA–RBP pairs were rigorously characterized by surface plasmon resonance (SPR). Two orthogonal synthetic RNA–RBP pairs with picomolar affinities were discovered, and one of them was also found to be orthogonal to the natural L7Ae–RNA complex.

## MATERIALS AND METHODS

### Materials

Unmodified oligo DNAs were purchased from Eurofins Genomics, FASMAC, or Integrated DNA Technologies (IDT) Japan. Randomized oligo DNAs containing NNK (where N = A, C, G, or T and K = G or T) codons were purchased from Ajinomoto Bio-Pharma or IDT Japan. DNA oligo pool was purchased from Twist Bioscience. Synthetic genes were purchased from Eurofins Genomics, IDT Japan, or Twist Bioscience. Biotinylated DNA and 2′-OMe RNA were purchased from FASMAC. Q5 High-Fidelity 2× Master Mix and HiScribe T7 High Yield RNA Synthesis Kit were purchased from New England Biolabs (NEB) for polymerase chain reaction (PCR) and in vitro transcription, respectively. SuperScript IV reverse transcriptase was purchased from Thermo Fisher Scientific for reverse transcription (RT). All chemicals used for buffer preparation were purchased from Nacalai Tesque or FUJIFILM Wako Pure Chemical Corporation. Difco LB broth Miller was purchased from BD for bacterial culture. *E. coli* Rosetta-gami B 5615 strain is not commercially available, because the manufacturer has discontinued production. Accordingly, we generated the strain in the laboratory as follows: pAR5615 plasmid DNA encoding isopropyl β-d-1-thiogalactopyranoside (IPTG)-inducible wild-type T7 phage capsid gene was purified from BLT5615 strain (Merck-Millipore) using Zyppy Plasmid Miniprep Kit (Zymo Research). The plasmid DNA was transformed into Rosetta-gami B competent cells (Merck-Millipore), and transformants were selected on an LB plate supplemented with antibiotics (50 μg/ml carbenicillin and 34 μg/ml chloramphenicol). MagStrep ‘type3’ XT beads (2-4090-002) was purchased from IBA Lifesciences. FG HM-NeutrAvidin beads (TAB8848N3171) and magnet stand (TAB4899N12) were purchased from Tamagawa SEIKI.

### Preparation of proteins for surface plasmon resonance (SPR) and electrophoretic mobility shift assay (EMSA)

Synthetic genes encoding L7Ae, dL7Ae, LS4, or LS12 with dual tags (N-terminal Twin-Strep-tag and C-terminal 6× His-tag) were subcloned into a pET vector backbone. The bacterial expression vectors (Supplementary Figure S1) were transformed into *E. coli* BL21 (DE3) strain. Transformants were pre-cultured overnight at 37°C in 2 ml of LB medium supplemented with 100 μg/ml carbenicillin and transferred to 100 ml fresh LB medium supplemented with 50 μg/ml carbenicillin. After 1–2 h culture at 30°C, IPTG was added at a final concentration of 0.4 mM, and the cells were further cultured for 20 h at 30°C. The cells were harvested by centrifugation at 6000 × *g* for 10 min at 4°C and suspended in 20 ml ice-cold His-tag purification buffer A (50 mM sodium phosphate, pH 7.7, 300 mM NaCl, 1 mM 2-mercaptoethanol, 5 mM imidazole). After cell disruption by ultrasonication, lysozyme (4 mg, Nacalai), Triton X-100 (0.1% v/v), MgCl_2_ (5 mM, NipponGene), DNase I (4 U, NEB), and RNase A (20 μg, NipponGene) were added to the crude cell extracts and incubated at 37°C for 30 min. The cell extracts were centrifuged at 8000 × *g* for 20 min at 4°C to remove cell debris, and the supernatants were filtered. Soluble proteins were loaded onto TALON metal affinity resin (TaKaRa) in a PD-10 (Cytiva) column and washed with 10 ml His-tag purification buffer A. His-tagged proteins were eluted by His-tag elution buffer (20 mM HEPES–KOH, pH 7.5, 200 mM NaCl, 1 mM 2-mercaptoethanol, 200 mM imidazole, 0.01% Tween 20 and 5% v/v glycerol), and the eluents were incubated with Strep-Tactin XT Superflow high capacity resin (IBA Lifesciences). After 5 min incubation, the resin was washed twice with 0.5 ml His-tag purification buffer A, and Twin-Strep-tagged proteins were eluted twice with 0.5 ml 1× Buffer BXT Strep-Tactin XT Elution Buffer (IBA Lifesciences). The eluents were desalted and concentrated using an ultrafiltration device (Amicon Ultra 0.5 ml filter, 3 kDa cut-off, Merck-Millipore) and buffer-exchanged into protein storage buffer (10 mM HEPES–KOH, pH 7.5, 140 mM KCl, 10 mM NaCl, 1 mM MgCl_2_, 0.1 mM TCEP-Na). Quality of the purified proteins was confirmed by SDS-PAGE (Mini-PROTEAN TGX gel system, Bio-Rad) followed by Coomassie blue staining (CBB Protein Safe Stain, TaKaRa) (Supplementary Figure S2). The concentrations of the recombinant proteins were determined using the standard curve generated from the band intensities of serially diluted bovine serum albumin (BSA) (Pierce Bovine Serum Albumin Standard Ampules, Thermo Fisher Scientific).

### Preparation of RNAs for SPR

Partially double-stranded DNAs or PCR products (46) were used as a template for *in vitro* transcription. RNA aptamers were synthesized by *in vitro* transcription at 42°C for 4 h using HiScribe T7 High Yield RNA Synthesis Kit (NEB) according to the manufacturer's manual for *in vitro* transcription of short templates (Supplementary Table S1). Transcription products were treated with 2 U TURBO DNase (Thermo Fisher Scientific) for 60 min at 37°C and purified with RNA Clean & Concentrator-25 Kit (Zymo Research). RNA concentrations were determined by absorbance at 260 nm according to OligoCalc (47).

### SPR

Binding kinetics were determined using Biacore T200 (Cytiva) at 25°C. CM5 chip surface was washed by three injections of 10 mM NaOH at a flow rate of 20 μl/min for 30 s. Next, a mixture of 200 mM 1-ethyl-3-(3-dimethylaminopropyl)carbodiimide hydrochloride (EDC) and 50 mM *N*-hydroxysuccinimide (NHS) was injected into all flow cells at a flow rate of 25 μl/min for 7 min to activate carboxylic acid groups. After injection of ultrapure water for 1 min, 0.3 mg/ml NeutrAvidin in 10 mM sodium acetate (pH 5.2) was injected by setting the immobilization level to 5000 RU. Finally, 1 M ethanolamine–HCl (pH 8.5) was injected for 7 min to quench the activated carboxylic acid. To remove the non-covalently bound NeutrAvidin on the chip surface, 10 mM NaOH was injected three times at a flow rate of 20 μl/min for 20 s. Setting the immobilization level to 400 RU, biotinylated 2′-OMe RNAs (5′-/bio/cguucugugucuuucgucgau-3′) (1 μM) in high salt buffer (10 mM Tris–HCl, pH 7.5, 1 M NaCl, 1 mM EDTA-Na) was injected and the remaining biotin-binding sites on the chip were quenched by injection of 1 μM free biotin.

RNA aptamer solutions (500 nM) were prepared in water and denatured by heating at 80°C for 3 min. After cooling at room temperature for few minutes, the RNA solutions were diluted to 10–50 nM with the high salt buffer. Various concentrations of protein solutions were prepared in SPR running buffer (10 mM HEPES, pH 7.4, 150 mM NaCl, 0.05% v/v Surfactant P20, 1 mM MgCl_2_, 0.25 mM TCEP-Na, 0.1 mg/ml BSA, 25 μg/ml yeast tRNA).

RNAs were injected at a flow rate of 10 μl/min for 30 s, and then the running buffer was injected for 1 min. Proteins were injected at a flow rate of 80 μl/min for 100 s (or 200 s) to monitor association, and then the dissociation kinetics was monitored for 420 s (or 1500 s) in SPR running buffer. The sensor surface was regenerated with 2 μl injection of 10 mM NaOH followed by 6 M urea at a flow rate of 20 μl/min and the running buffer was injected for 1 min. The raw data were analyzed by Biacore T200 Evaluation Software 1.0 (Cytiva) using 1:1 Langmuir interaction model. Background signal from the reference flow cell was subtracted from that of the sample flow cell, and no-protein sample (SPR running buffer only) was injected in each experiment (double referencing). Dissociation constant (*K*_D_) was determined from the ratio of the association and the dissociation rate constants (*K*_D_*= k*_off_ / *k*_on_). The figures were generated using GraphPad Prism software 6.0h (GraphPad Software). The measurements were repeated at least twice using different flow cells (or different SPR chips) in different days to ensure reproducibility.

### Preparation of affinity beads

MagStrep ‘type3’ XT beads were rinsed three times with ultrapure water and stored in blocking buffer (Pierce Protein-Free TBS Blocking Buffer, Thermo Fisher Scientific) at 4°C. FG HM-NeutrAvidin beads (100 μl) were rinsed once with immobilization buffer (10 mM Tris–HCl, pH 7.5, 1 M NaCl, 0.1 mM EDTA-Na), and mixed with 1.64 nmol of biotinylated capture oligo DNA (5′-/bio/CGTTCTGTGTCTTTCGTCGAT-3′) in the same buffer and incubated at room temperature for 5 min. After washing with the same buffer three times, FG beads were stored in blocking buffer at 4°C. The immobilized oligo DNA was estimated to be ∼6.3 pmol per μl suspended beads. The beads were pre-equilibrated with selection buffer (10 mM HEPES–KOH, pH 7.5, 140 mM KCl, 10 mM NaCl, 1 mM MgCl_2_, 0.01% Tween 20, 0.1 mM TCEP-Na, 2% v/v glycerol) before use.

### Construction of PD library

*A. fulgidus* rpl7ae gene was sub-cloned into T-vector pMD20 (TaKaRa), and the sequence was verified by Sanger sequencing. The partially randomized DNA library encoding L7Ae mutants was prepared by overlap extension PCR (48) as summarized in Supplementary Figure S3. Briefly, three fragments outside of the randomized regions were amplified by PCR using appropriate primer pairs (EcoRI-T7PD_Fw and L7Ae-Fr1_Rv, L7Ae-Fr2_Fw and L7Ae-Fr2_Rv, L7Ae-Fr3_Fw and HindIII-T7PD_Rv; Supplementary Table S2). Next, two of these fragments were used as templates to add degenerate codons (NNK) at the targeted positions in L7Ae using appropriate primers (L7Ae-Fr2_lib_Fw and L7Ae-Fr2_Rv, L7Ae-Fr3_lib_Fw and HindIII-T7PD_Rv; Supplementary Table S2). Finally, three DNA fragments containing overlap regions were assembled by overlap extension PCR using primers EcoRI-T7PD_Fw and HindIII-T7PD Rv (Supplementary Table S2). The final PCR product was digested with Eco RI-HF (NEB), Hind III-HF (NEB), Dpn I (NEB), and gel purified using Zymoclean Gel DNA Recovery Kit (Zymo Research). Sequence of the assembled DNA library was confirmed by Sanger sequencing (Supplementary Figure S3). L7Ae-scaffold T7 PD library was constructed using T7Select10-3b system (Merck-Millipore, 70550–3CN). The digested DNA library (∼0.7 pmol) was ligated with T7Select10-3b vector arms (5 μg) with 400 U T4 DNA ligase (NEB) at 16°C for 16 h in 20 μl scale. After addition of 125 μl of T7 packaging extract, in vitro DNA packaging reaction was performed at 22°C for 2 h and quenched by addition of LB medium. Diversity of the PD library was estimated by plaque assay (∼4.0 × 10^8^). The PD library was amplified with logarithmic phase of Rosetta-gami B 5615 strain in 200 ml scale (multiplicity of infection < 0.01). Expression of Gp10A was induced in advance by addition of 1 mM IPTG. Upon lysis (usually 1.5–2 h after infection), 5 g of sodium chloride was added to the lysate and the *E. coli* debris was removed by centrifugation at 8000 × *g* for 20 min at 4°C. After collection of the supernatant, 21 g of polyethylene glycol (PEG) 8000 (Promega) was added and incubated at 4°C overnight. Lysate-PEG mixture was centrifuged at 8000 × *g* for 40 min at 4°C and the supernatant was discarded. The precipitated phage particles were suspended in T7 phage stock buffer (10 mM Tris–HCl, pH 8.0, 1 M NaCl, 1 mM EDTA-Na), and subjected to ultrasonication for few minutes. The phage solution was filtered through a 0.45 μm membrane filter (hydrophilic polytetrafluoroethylene, Merck-Millipore), and stored at 4°C until use. Titer of the amplified phage was quantified by plaque assay. Bacteriophage T7-displayed L7Ae or dL7Ae was confirmed by Western blotting (Supplementary Figure S4). The amino acid distributions of the randomized residues were analyzed by Illumina sequencing (Supplementary Figure S5). We found overrepresentation of amino acids arginine, leucine, serine and other amino acids as would be expected from the NNK degenerate codons.

### Construction of RNA library

The template DNA was prepared by primer extension as described previously with modifications (49). Two oligo DNAs (200 pmol each of N20L_Fw and N20L_lib_Rv; Supplementary Table S2) were mixed with Q5 High-Fidelity Master Mix (NEB) in 200 μl scale, and the mixture was denatured at 98°C for 1 min. The oligos were annealed and extended at 72°C for 2 min. The product was analyzed by 2% agarose gel electrophoresis and purified with DNA Clean & Concentrator Kit (Zymo Research). Approximately 16.7 pmol (1.0 × 10^13^ molecules) of the purified DNA was used as a template in an in vitro transcription reaction at 37°C for 4 h in 200 μl volume (0.75× reaction buffer, 7.5 mM NTPs, 40 U Murine RNase inhibitor, 7.5 μl of T7 RNA polymerase mix). The transcription product was treated with 2 U TURBO DNase (Thermo Fisher Scientific) at 37°C for 60 min and purified by phenol-chloroform extraction followed by ethanol precipitation. Purified RNAs were dissolved in 1× RNA loading dye (NEB), heat-denatured at 80°C for 3 min, and separated by 8% PAGE. The separated sample was visualized by UV shadowing to excise the gel fragment and the RNA was eluted overnight in TE buffer. The eluents were filtered through a 0.22 μm membrane filter (hydrophilic polytetrafluoroethylene, Merck-Millipore), and concentrated using an ultrafiltration device (Amicon Ultra 0.5 ml filter, 3 kDa cut-off, Merck-Millipore). RNA concentration was determined from absorbance at 260 nm using NanoDrop One (Thermo Scientific).

### PD-SELEX

A detailed summary of the selection parameters is shown in Supplementary Table S3. In the 1st purification, an appropriate titer of the PD pool was mixed with 1/5 volume of 20% PEG-8000, 2.5 M NaCl solution and precipitated by centrifugation at 18 000 × *g* for 10 min at 4°C. The phage particles were dissolved in 500 μl selection buffer and was incubated with 10 μl pre-equilibrated MagStrep ‘type3’ XT beads at room temperature for 30 min. The phage-immobilized beads were washed once with 500 μl selection buffer and re-suspended in 295 μl of the same buffer. RNA solution of an appropriate concentration was prepared in 200 μl volume, and the RNAs were folded by heating at 80°C for 3 min followed by incubation at room temperature. The folded RNAs were mixed with 4 μl yeast tRNA (40 μg, Ambion), 40 U Murine RNase Inhibitor (NEB), and 395 μl of the phage-immobilized magnetic beads. After washing with selection buffer, phage particles were eluted with 50 μl 1× Buffer BXT Strep-Tactin XT Elution Buffer (100 mM Tris–HCl, pH 8.0, 150 mM NaCl, 1 mM EDTA-Na, 50 mM biotin) (IBA Lifesciences).

In the 2nd purification, the eluent from the 1st purification was mixed with 10 μl of capture oligo DNA-immobilized NeutrAvidin beads and 440 μl selection buffer and incubated. After washing with the same buffer, the beads were re-suspended in 20 μl of TE buffer. The bead suspension was split into two aliquots for separate RNA and phage recovery. To recover RNAs, 20 pmol of reverse primer (N20L_Rv, Supplementary Table S2) was added to the suspension and incubated at 80°C for 3 min. The beads were separated by a magnet and the supernatant was collected and kept on ice. To recover the phage particles, 60 U RNase H (TaKaRa) and 5× RNase H reaction buffer (250 mM Tris–HCl, pH 7.5, 375 mM NaCl, 15 mM MgCl_2_, 5 mM dithiothreitol) were added to another aliquot of the beads and incubated at 30°C for 15 min. The beads were separated by a magnet and the supernatant containing phage particles was collected.

The recovered RNAs were reverse transcribed at 60°C for 15 min in 20 μl volume using SuperScript IV, and the reaction was quenched by incubation at 85°C for 5 min. The cDNAs were amplified by PCR (20 cycles of 98°C for 10 s, 71°C for 20 s, 72°C for 10 s) with appropriate primers (N20L_Fw and N20L_Rv, Supplementary Table S2). The PCR products were purified using Zymoclean Gel DNA Recovery Kit, and 75% of the purified DNA was used for in vitro transcription (20 μl scale) to produce the RNA pool for the next round. In vitro transcription products were treated with 2 U TURBO DNase (Thermo Fisher Scientific) at 37°C for 60 min and purified with RNA Clean & Concentrator-25 Kit. The recovered phage particles were amplified in 35 ml scale, and the amplified phage particles were purified by the PEG-NaCl method followed by filtration with a 0.45 μm membrane filter.

### Illumina sequencing and data analysis

Illumina sequencing libraries were prepared by RT-PCR (for RNA library) or two-step PCR (for PD library). The RNAs were mixed with a barcoded reverse transcription primer (N20L_MiSeq_bc_RT, Supplementary Table S2) and denatured at 80°C for 3 min. After cooling on ice, reverse transcription was carried out at 60°C for 15 min. The cDNA was amplified by 10 cycles of PCR using appropriate primers (N20L_MiSeq_P7_F and MiSeq_R1seq_P5_R, Supplementary Table S2).

The phage genomic DNAs were amplified by 6 cycles of PCR using barcoded primers (R1_bc_LS_Fw and R2_LS_Rv, Supplementary Table S2), and the amplified DNAs were purified with DNA Clean & Concentrator Kit. The purified first PCR products were further amplified by 10 cycles of PCR using appropriate primers (P5_R1_Fw and P7_R2_Rv, Supplementary Table S2) to add P5 and P7 adapter sequences.

The barcoded Illumina libraries were separated by 3% agarose gel and purified with Zymoclean Gel DNA Recovery Kit. The purified libraries were quantified by real-time PCR using NEBNext Library Quant Kit for Illumina (NEB) according to manufacturer's instructions. PhiX control v3 DNA (10%, Illumina) was spiked into the libraries as an internal control, and sequencing was performed on Illumina MiSeq sequencer using MiSeq Reagent Kit v3 (150 cycle single read or 300 cycle paired-end read). Raw sequence data (fastq files) were analyzed and visualized by custom Python scripts.

### Construction of second-generation RNA library

The oligo pool (426 fmol) containing 2000 distinct sequences was amplified by PCR (25 cycles of 98°C for 10 s, 70°C for 20 s, 72°C for 10 s) using N20L_Fw and N20L_Rev (Supplementary Table S2) as primers. The PCR product was purified using Zymoclean Gel DNA Recovery Kit, and the purified DNA (18.7 pmol) was used for in vitro transcription in 100 μl volume to obtain the second-generation RNA library. Transcription products were treated with 2 U TURBO DNase (Thermo Fisher Scientific) and 20 U Exonuclease I (NEB) at 37°C for 30 min and purified with RNA Clean & Concentrator-25 Kit.

### Preparation of affinity beads for re-selection

Bacterial expression vectors (Supplementary Figure S1) were introduced into *E. coli* BL21 (DE3) strain. The transformants were cultured overnight at 30°C in 2 ml of MagicMedia Medium (Thermo Fisher Scientific) supplemented with 50 μg/ml carbenicillin. The cells were harvested and suspended in ice-cold His-tag purification buffer B (20 mM HEPES–KOH, pH 7.5, 200 mM NaCl, 1 mM 2-mercaptoethanol, 1 mM imidazole, and protease inhibitor cocktail without EDTA). After cell disruption by ultrasonication, Triton X-100 (0.05% v/v) was added to the crude cell extract. The extract was centrifuged at 18 000 × *g* for 10 min at 4°C to remove cell debris. The supernatant was incubated with TALON metal affinity resin (30 μl, Clonetech) for 10 min at 4°C. After washing several times with the same buffer, His-tagged protein was eluted with His-tag elution buffer. His-tag purified proteins were confirmed by Western blotting with Strep-Tactin-HRP conjugate (1:10 000 dilution, Bio-Rad) (Supplementary Figure S6).

MagStrep ‘type3’ XT beads (10 μl, Clonetech) were rinsed three times with ultrapure water and then incubated with blocking buffer (Pierce Protein-Free TBS Blocking Buffer, Thermo Fisher Scientific) at 4°C overnight. His-tag purified proteins were incubated with 10 μl of MagStrep ‘type3’ XT beads at 4°C for 30 min. After washing with 500 μl each of high salt buffer (10 mM Tris–HCl, pH 7.5, 500 mM NaCl, 1 mM EDTA-Na) and tTBS (25 mM Tris–HCl, pH 7.4, 137 mM NaCl, 27 mM KCl, 0.05% Tween 20) (Nacalai), the beads were stored in blocking buffer at 4°C.

### Re-selection of second-generation RNA pool

RNA solution (0.2 μM) was prepared in 500 μl selection buffer supplemented with 0.5 M urea, and the RNAs were folded by heating to 80°C for 3 min followed by incubation at room temperature. The folded RNAs were mixed with 1 μl yeast tRNA (10 μg, Ambion) and 5 μl recombinant protein-immobilized beads, and incubated at 37°C for 30 min. After washing eight times with 500 μl sample buffer, RNAs were eluted with 50 μl 1× Buffer BXT Strep-Tactin XT Elution Buffer (IBA Lifesciences). The eluents were purified and concentrated by ethanol precipitation and then dissolved in 10 μl TE.

## RESULTS

### Design of PD-SELEX

We designed the following strategy to enrich complexes of RNA and phage-displayed protein from unbound species via two-step affinity purification (Figure 1). First (Figure 1, 1st purification), phage particles are immobilized on Strep-Tactin magnetic beads via Strep-tag II (50) which is fused to the C-terminus of the phage-displayed protein. The RNA pool is added to the phage-immobilized magnetic beads and allowed to form RNA–RBP complexes. Then the beads are washed to remove unbound RNAs. The phage particles (bound and unbound to RNA) are recovered from the beads by competitive elution with D-biotin. In the second step (Figure 1, 2nd purification), phage-bound RNAs are captured by DNA-immobilized NeutrAvidin magnetic beads. The DNA sequence is complementary to the fixed sequence at the 3′ end of the RNA library (docking sequence). It should be noted that the Strep-tag II fused to RBP does not bind to NeutrAvidin (51,52). The beads are then washed to remove unbound phage particles.

Through this two-step affinity purification protocol, complexes of RNA and phage-displayed protein are enriched. The beads are split into two aliquots to recover the enriched RNAs and phage particles separately. RNAs and phage particles are recovered by heating and ribonuclease H (RNase H) treatment, respectively. As RNase H cleaves the RNA strand in DNA–RNA hybrid, it helps to reduce phage particles that non-specifically bind to the capture DNA, NeutrAvidin, or magnetic beads. The recovered RNAs are amplified by reverse transcription (RT) followed by polymerase chain reaction (PCR) and in vitro transcription (IVT). The recovered phage particles are used to infect *E. coli* to be amplified. The amplified RNA and phage pools are then used for the subsequent round of selection. After several rounds of selection, the enriched RNA and phage-displayed RBP sequences are analyzed by next-generation sequencing (NGS).

### PD-SELEX mock selection

**Figure 2.**
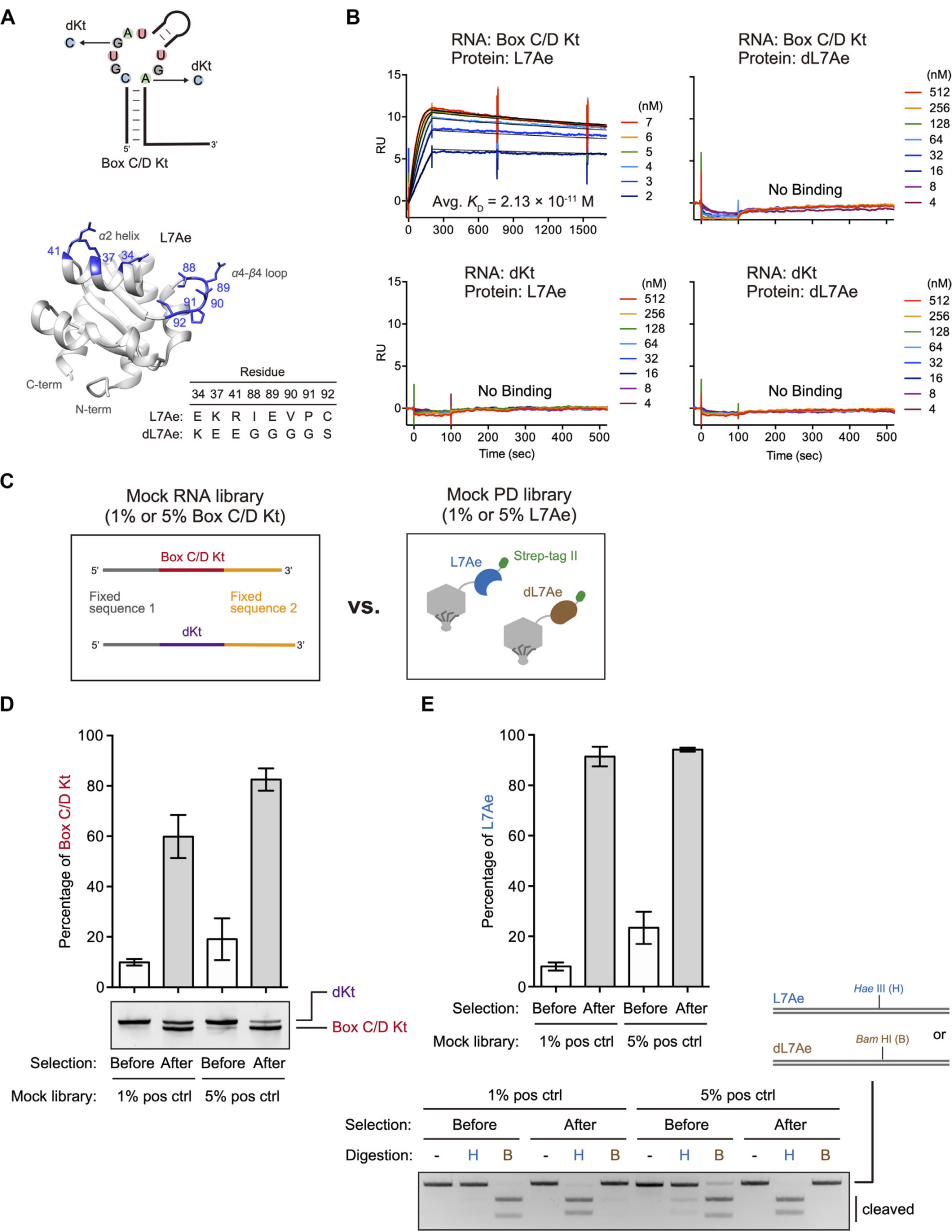
Mock selection. (**A**) Schematic representation of the box C/D Kt and dKt (defective Kt) RNAs, and *A. fulgidus* L7Ae structure (PDB ID, 4BW0) (54) depicting the mutated residues in dL7Ae. The graphic was generated using UCSF Chimera (71). (**B**) Confirmation of binding specificity of the RNAs (box C/D Kt or dKt) and the recombinant proteins (L7Ae or defective L7Ae: dL7Ae) by SPR. Sensorgrams are shown in colored lines, and black lines indicate curve fitting according to 1:1 binding model. RU stands for response unit. The observed kinetic values and dissociation constants are summarized in Table 1. For box C/D Kt–L7Ae, dissociation time was extended to 25 min from 7 min to monitor slow dissociation. (**C**) Schematic illustration of the mock libraries. (**D**) Mock RNA library selection. RT-PCR products (20 ng) were separated by native PAGE and visualized by SYBR Gold staining. The sizes of the box C/D Kt and dKt are 91 bp and 96 bp, respectively. The band intensities were quantified using ImageJ. The bar graph represents means and standard deviations of three independent experiments. (**E**) Mock PD library selection. PCR products (100 ng) were digested with a restriction enzyme Hae III (H) or Bam HI (B), and then separated by 2% agarose gel electrophoresis. DNAs were visualized by ethidium bromide staining. The band intensities were quantified using ImageJ, and the bar graph represents means and standard deviations of three independent experiments.

To establish the PD-SELEX protocol described above, we designed and executed a mock selection using the natural box C/D Kt and L7Ae (32) as a model RNA–RBP pair, and a defective k-turn (dKt) RNA with no affinity to L7Ae (42) as a negative control RNA. Additionally, we designed dL7Ae, a non-functional variant of *A. fulgidus* L7Ae that lacks affinity for box C/D Kt as a negative control protein (Figure 2A). Three charged amino acids (E34, K37, R41) in the *α*2 helix of L7Ae are evolutionarily conserved in archaea to eukaryotes (53). It is known that K37 and R41 interact with the phosphates of Watson-Click base pairs in Kt RNA, and E34 interacts with guanine in the G•A pair adjacent to the kinked buldge in Kt RNA (54). We substituted three of these charged amino acids with oppositely charged residues (E34K, K37E, R41E). The amino acid sequence of the loop connecting the *α*4 helix and *β*4 sheet is not conserved between archaea and eukaryotes (53), and three residues I88, E89, and V90 of *A. fulgidus* L7Ae recognize nucleobases in the bulge region of Kt RNA (54). Therefore, we also substituted five amino acids in the loop to glycine or serine (I88G, E89G, V90G, P91G, C92S). dLA7e, with 8 amino acid substitutions (E34, K37, R41, I88G, E89G, V90G, P91G, C92S) lost binding affinity to box C/D Kt and did not bind to dKt as measured by SPR at least within the range of measured protein concentrations (Figure 2B). We also confirmed that L7Ae does not bind to dKt which is consistent with the previous report (42). The observed dissociation constant (*K*_D_) between L7Ae and box C/D Kt was 2.13 × 10^–11^ M in physiological salt and magnesium concentrations at pH 7.4 (Figure 2B and Table 1). This is comparable to the reported *K*_D_ value of 10 pM between L7Ae and another Kt RNA (ribosomal Kt-7) measured by stopped-flow kinetic analysis (55).

**Table 1.** Binding properties of model RNAs and proteins determined by SPR.

RNA	Protein	*k*_on_ (M^–1^ s^–1^)	*k*_off_ (s^–1^)	*K*_D_ (M)
Box C/D Kt	L7Ae	(1.41 ± 1.11) × 10^7^	(2.77 ± 1.75) × 10^–4^	(2.13 ± 0.43) × 10^–11^
	dL7Ae	N.B.	–	–
dKt	L7Ae	N.B.	–	–
	dL7Ae	N.B.	–	–

*k*_on_, *k*_off_, *K*_D_ values are means and standard deviations of at least two independent experiments.

N.B.: no binding detected at the protein concentrations tested.

Next, we generated T7 phage particles displaying L7Ae or dL7Ae with a C-terminal Strep-tag II using the T7Select10-3b system (Figure 2C), and the protein expression was confirmed by Western blotting (Supplementary Figure S4). Also, box C/D Kt and dKt RNA sequences flanked by fixed regions (Figure 2C) were prepared by in vitro transcription. Mock PD libraries were prepared by mixing phage particles displaying L7Ae and dL7Ae at 1:99 or 5:95 ratio. Similarly, box C/D Kt and dKt RNAs were mixed in 1:99 or 5:95 ratio to prepare mock RNA libraries. As illustrated in Figure 1, 1 × 10^11^ plaque forming unit (PFU) of a mock PD library was immobilized on StrepTactin magnetic beads. Next, the beads were incubated with 0.2 μM mock RNA library in 500 μl buffer for 10 min, and the beads were washed three times with the same buffer. The phage particles and the RNAs were eluted with D-biotin and then incubated with DNA-immobilized NeutrAvidin magnetic beads in 500 μl of buffer for 10 min to capture RNA-phage complexes. After washing three times, RNAs were eluted, reverse transcribed, and amplified by PCR. Since dKt was designed to be 5-nt longer than box C/D Kt, relative abundance of the two RNA species could be evaluated by gel electrophoresis of the PCR products (Figure 2D). Box C/D Kt RNA was enriched 6.0-fold or 4.3-fold after a single round of selection as estimated from the intensities of the two bands (Figure 2D). Similarly, the mutated region of the L7Ae/dL7Ae proteins was PCR amplified from the phage genomic DNA before and after the mock selection. The PCR products were then digested with Hae III or Bam HI which specifically cleaves L7Ae or dL7Ae PCR products, respectively (Supplementary Figure S7). Again, 11.4-fold or 4.0-fold enrichment of L7Ae was observed after a single round of mock selection (Figure 2E). These results demonstrate that the two-step affinity purification protocol efficiently enriches RNA–RBP pairs from the background of non-binding RNAs and RBPs.

### Library-vs-library selection of RNA–RBP pairs

**Figure 3.**
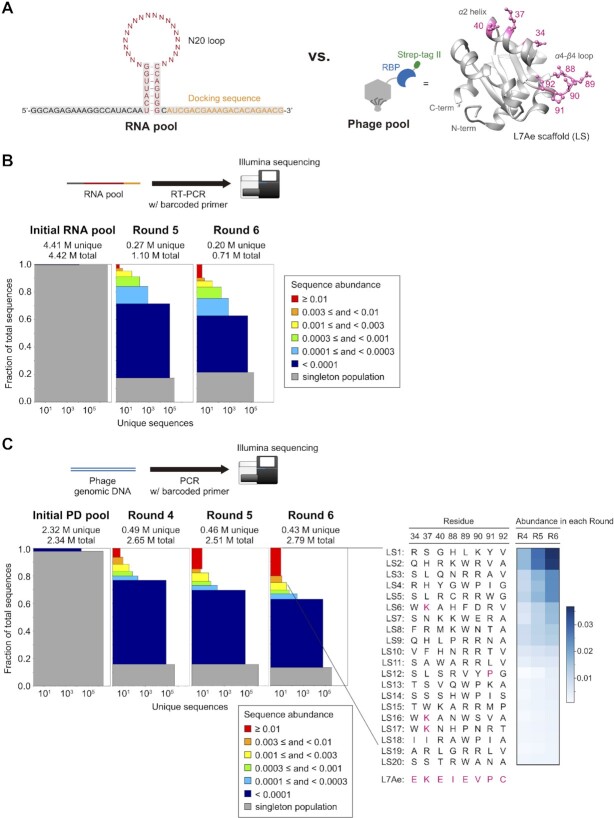
RNA library vs. PD library in vitro selection. (**A**) Design of the hairpin N20-loop RNA library (left, N = A, C, G or U) and L7Ae-scaffold protein library (right). Randomized amino acid residues are shown in pink. Population diversity (72,73) of the initial and the selected (**B**) RNA pool and (**C**: left) PD pool. Multi-copy sequences were sorted into six colored bins according to abundance. Single-count sequences are shown in the bottom grey bins (singleton population). The height of each bin represents its proportion in the population. The width represents the number of unique sequences. (**C**: right) Amino acid sequences of LS1-LS20. The amino acid residues found in L7Ae are shown in pink. The heatmap shows the abundance of each LS variant in rounds 4, 5 and 6.

After establishing the selection protocol, we set out to perform library-vs-library selection of novel RNA–RBP pairs. We designed a hairpin loop RNA library in which 20-nt in the loop region were randomized which contains up to 1.1 × 10^12^ unique sequences (Figure 3A, left) with an expectation that structured RNA pools contain higher frequency of RNA aptamers (56). Fixed sequences were added to the 5′ and 3′ ends of the hairpin loop library for amplification and affinity purification. *A. fulgidus* L7Ae was used as a scaffold for the RBP library. Guided by the reported crystal structure (54), three charged residues in the *α*2 helix (E34X, K37X, E40X, where X stands for 20 amino acids) and five residues (I88X, E89X, V90X, P91X, C92X) in the loop connecting *α*4 helix and *β*4 sheet were randomized. We did not randomize R41 because it is considered to form a universal hydrogen bond with a phosphate group in Kt RNA (57) which does not appear to be involved in RNA sequence selectivity. Although the theoretical diversity of the L7Ae scaffold (LS) PD library is 2.56 × 10^10^ ( = 20^8^), diversity of the actual library is limited by the efficiency of in vitro packaging reaction of the phage genomic DNA which was estimated to be approximately 4.0 × 10^8^ based on plaque assay. The PD library size is comparable to those of the previously reported T7 PD libraries (20,58,59). Fewer amino acids should be randomized if complete or near-complete coverage of the amino acid combinations is desired. We opted to randomize more residues than can be fully represented in the PD library because our goal was to discover novel RBPs with new RNA sequence selectivity, and we did not know which residues are more important for recognizing new RNA motifs yet to be discovered.

Six rounds of library-vs-library selection were carried out. Stringency of the selection was gradually increased by lowering the amounts of RNA and PD pools used, shortening the binding time, and increasing the number of washing steps (Supplementary Table S3). In the first two rounds, negative selections were performed to avoid enrichment of non-specific variants that bind to magnetic beads and/or phage particles. In the T7Select10-3b system used for PD library construction, roughly 10 copies of proteins are displayed on each virion which could increase the apparent affinity to the immobilized targets due to multivalency. Therefore, in the last two rounds, we added 0.5 M urea as a denaturant to the binding/washing buffer to increase the selection pressure.

After eluting the RNA and the PD pools, the initial and the selected sequences after rounds 4 (PD only), 5 and 6 were analyzed by Illumina sequencing. As expected, almost all sequences in the initial RNA and PD pools (99.8% of RNA and 99.2% of PD) were sequenced only once (Figure 3B and C). RNA and protein sequences were enriched through the selection (Figure 3B and C), and significant enrichment of both RNA and phage populations was observed prior to the 6th round. For each protein sequence, enrichment factor (EF) was calculated by dividing the abundance of the sequence after round N by that of round N-1. After filtering the protein sequence data to keep those that are enriched from rounds 4 through 6 (EFs from round 4 to 5 and round 5 to 6 ≥ 1.0), the 20 most abundant variants in round 6 of the PD pool (LS1-LS20) were selected for further analysis (Figure 3C). The enriched LS proteins showed no similarity to the parental L7Ae (Figure 3C) and the archaeal L7Ae homologs (Supplementary Figure S8A). However, some sequence trends were found among the laboratory-evolved proteins (Supplementary Figure S8B). For example, tryptophan or arginine was preferred in the 89th residue in the 20 most abundant sequences. Preference for arginine at residue 89 (but not tryptophan) was still observed when the top 1000 sequences were examined. Similarly, arginine was frequently observed in the 90th residue in both top 20 and top 1000 sequences. No strong preferences at other positions were observed (Supplementary Figure S8B).

### Deconvolution of RNA–LS protein pairs

**Figure 4.**
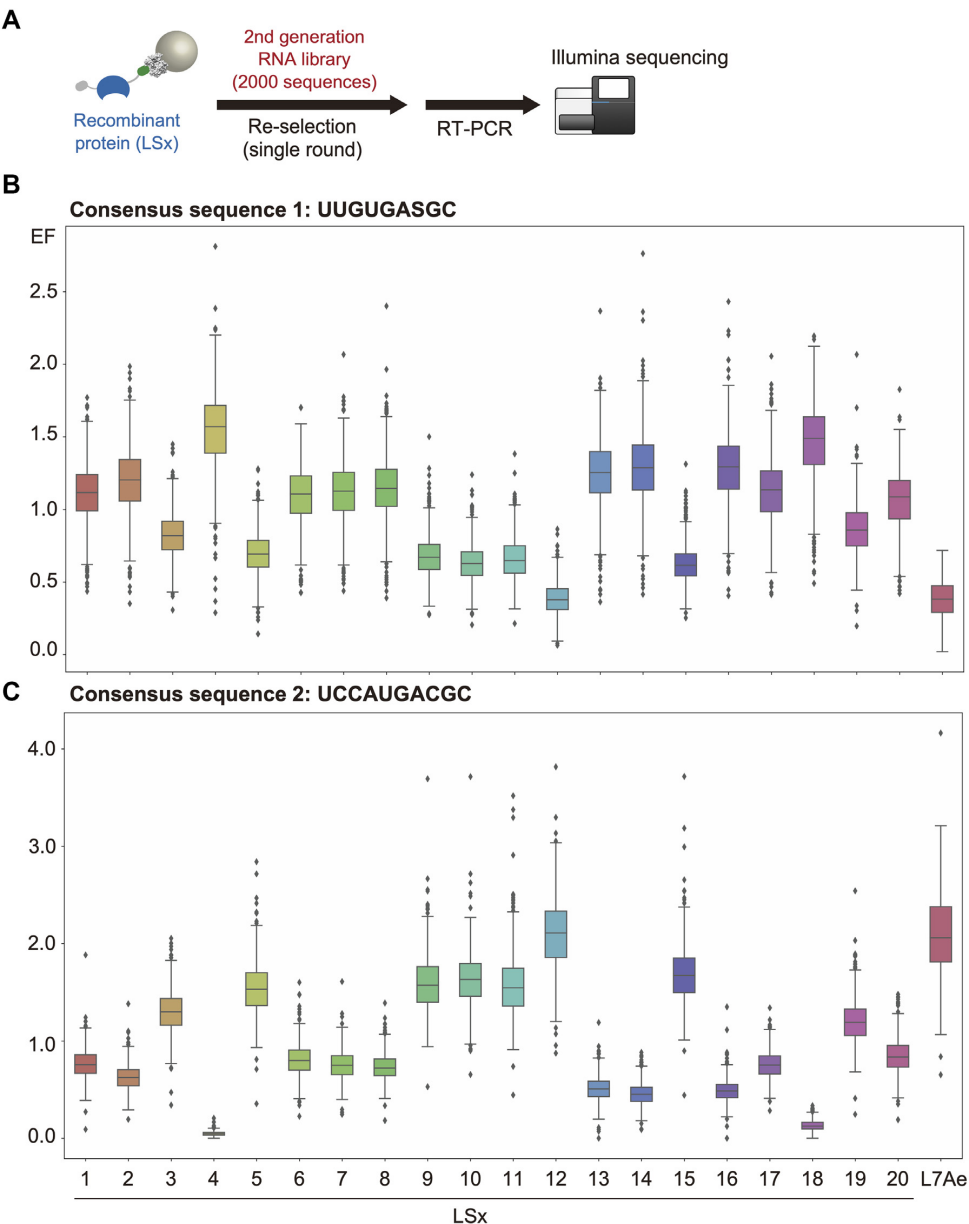
Re-selection of the second-generation RNA library against individual L7Ae mutants (LS1-LS20) and L7Ae. (**A**) Schematic illustration of the re-selection experiment. A focused RNA library containing the 1991 most abundant RNAs after PD-SELEX and 9 control RNAs was selected against individual L7Ae or its mutant immobilized on magnetic beads. (**B**, **C**) Box plots representing enrichment factors (EFs) of a subset of the RNAs that contains consensus sequence 1 (UUGUGASGC, S = C or G) (**B**) or consensus sequence 2 (UCCAUGACGC) (**C**). EF is defined as the abundance of an RNA after selection divided by the abundance before selection.

Sequencing of the enriched RNA and LS protein sequences does not indicate which RNA interacts with which LS protein. To discover specific RNA–LS protein pairs, we prepared recombinant LS1–LS20 and L7Ae proteins and immobilized them individually on magnetic beads. We also synthesized the 1991 most abundant RNA sequences after the 6th round and 9 control RNAs (L7Ae-binding RNAs: box C/D Kt, Kt-7, Kt-15, lysine riboswitch Kt, SAM riboswitch Kt, H/ACA sRNA loop, sR8 C’/D’ box loop, H23 aptamer, and negative control: dKt) (13,42) by in vitro transcription from a dsDNA template prepared from a custom oligo pool. The 2000-sequence RNA library was selected for binding against each immobilized LS protein (and L7Ae) with stringent binding and washing steps (Figure 4A). The eluted RNAs were reverse transcribed with a barcoded primer and PCR amplified for Illumina sequencing. For each RNA, an EF was calculated as: EF = abundance after selection / abundance before selection.

A simple inspection of the RNA sequences after PD-SELEX revealed two highly enriched consensus sequences. Consensus sequence 1 (UUGUGASGC, S = C or G) was found in 57% of the population, and consensus sequence 2 (UCCAUGACGC) was found in 25% of the population after round 6. The 2000-sequence RNA library also contained 59% and 33% of the consensus sequence 1 and 2, respectively. In contrast, the unselected pool contained 0.0109% (consensus sequence 1) and 0.0011% (consensus sequence 2) of the two sequences. Consequently, we focused on binding of these consensus sequences to the individual LS proteins.

Enrichment of the RNAs possessing consensus sequence 1 or 2 was statistically analyzed and visualized by box plots (Figure 4B and C). Judging from the median values, RNAs containing consensus sequence 1 were significantly enriched by LS4 and LS18 (Figure 4B). Interestingly, LS4 and LS18 share the same subsequence in the loop region (W89, P90, and I91), suggesting that these residues contribute to the affinity for consensus sequence 1 (Figure 3C). In the case of consensus sequence 2, the RNAs were significantly enriched by LS5, LS9, LS10, LS11, LS12, LS15 and L7Ae (Figure 4C), and among them, LS5, LS9, LS10, LS11 and LS15 share the same amino acids at two positions (R89 and R90) (Figure 3C) while the 91st residue of LS12 is the same as that of L7Ae (P91).

Further inspection revealed that LS4 has low EF values (<0.5) for consensus sequence 2, and LS12 and L7Ae have low EF values (<0.5) for consensus sequence 1 (Figure 4B and C). This suggests that LS4 may selectively bind RNA sequences containing consensus sequence 1, and LS12 and L7Ae may selectively bind RNA sequences containing consensus sequence 2, therefore could function as orthogonal RNA–RBP pairs.

### Binding affinities of LS4 and LS12 with selected RNAs

**Figure 5.**
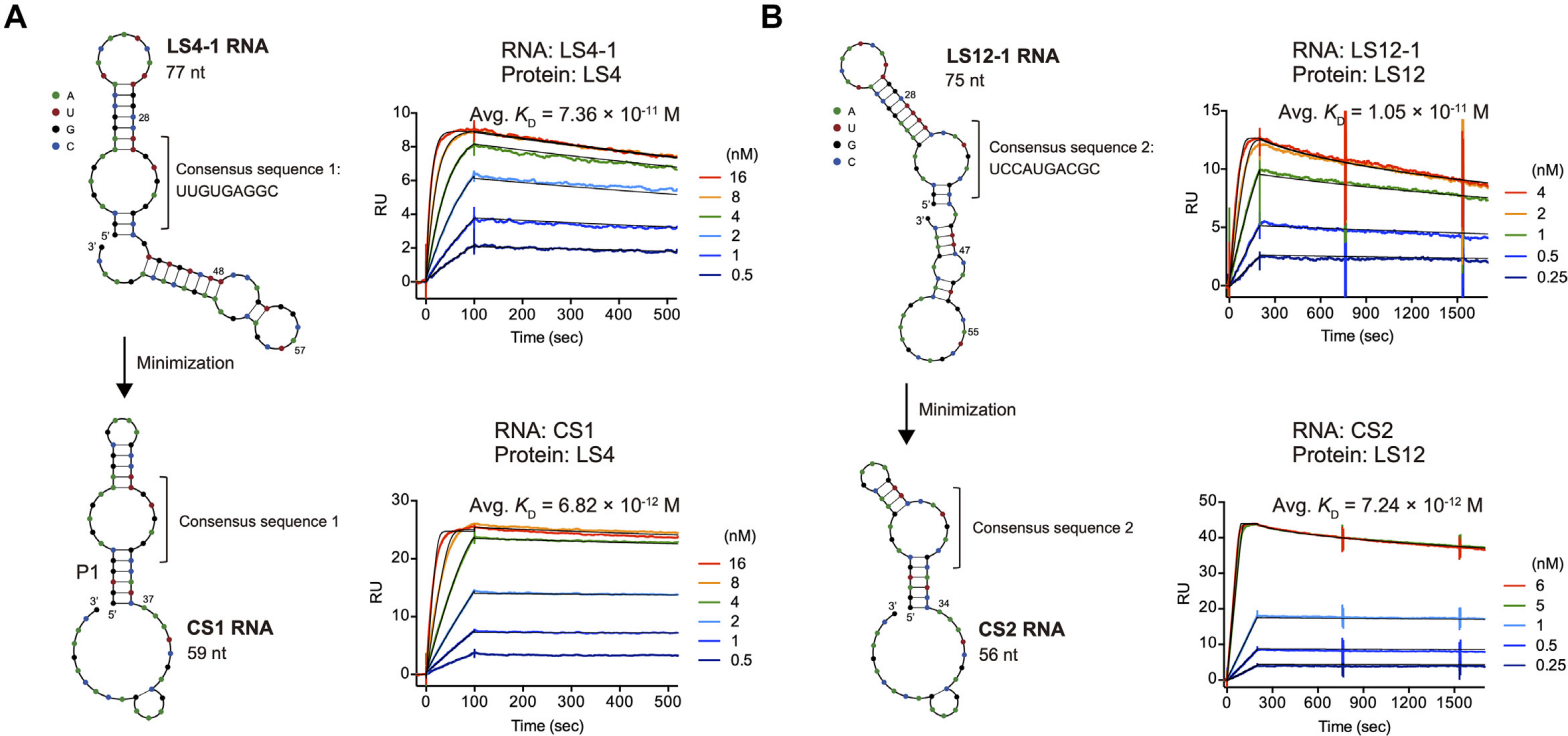
Characterization of binding affinity between the selected RNAs and RBPs. (**A**) SPR sensorgrams of LS4-1 RNA and LS4 protein (top), and the minimized CS1 RNA and LS4 protein (bottom). (**B**) SPR sensorgrams of LS12-1 RNA and LS12 protein (top), and the minimized CS2 RNA and LS12 protein (bottom). Observed kinetic values and dissociation constants are summarized in Table 2. It should be noted that 3′ end of the minimized RNAs (CS1: bases 37–59, CS2: bases 34–56) were used for immobilization on the SPR chip.

We chose RNA sequences that exhibited the highest EF value for LS4 or LS12 (EF of LS4-1 = 2.8; EF of LS12-1 = 3.8) and measured their binding affinities using SPR (Figure 5). RNAs were captured on a 2′-OMe RNA-immobilized sensor chip, and varying concentrations of proteins were injected to determine the binding and dissociation kinetics. LS4-1 RNA bound LS4 with a *K*_D_ of 7.36 × 10^–11^ M, and LS12-1 RNA bound LS12 with a *K*_D_ of 1.05 × 10^–11^ M (Figure 5 and Table 2). Secondary structure prediction by NUPACK (60) suggested that the consensus sequence 1 in LS4-1 RNA likely formed an internal loop as depicted in Figure 5A. We hypothesized that this internal loop structure plays a critical role for binding to LS4 and designed CS1 RNA (Figure 5A), a minimized version of LS4-1 RNA. Strikingly, CS1 RNA bound to LS4 with a dissociation constant of 6.82 × 10^–12^ M, approximately 10-fold improvement relative to LS4-1 RNA presumably due to the stabilization of the P1 stem. Similarly, the consensus sequence 2 was predicted to form an asymmetric internal loop in LS12-1 RNA (Figure 5B) which comprises a putative kink-turn motif (61). Accordingly, LS12-1 RNA was minimized to CS2 RNA (Figure 5B) whose *K*_D_ for LS12 binding was measured to be 7.24 × 10^–12^ M which is comparable to the that of the parental RNA. Electrophoretic mobility shift assay (EMSA) of CS1 RNA–LS4 and CS2 RNA–LS12 using 0.5 nM Cy5-labeled RNA showed binding profiles characteristic of titration regime (62) which indicates *K*_D_ << 5 × 10^–10^ M (Supplementary Figure S9).

**Table 2.** Binding characteristics of the RNA–RBP pairs from PD-SELEX

RNA	Protein	*k*_on_ (M^–1^ s^–1^)	*k*_off_ (s^–1^)	*K*_D_ (M)
LS4-1	LS4	(6.94 ± 0.60) × 10^6^	(5.06 ± 0.56) × 10^–4^	(7.36 ± 1.43) × 10^–11^
CS1		(2.00 ± 0.07) × 10^7^	(1.36 ± 0.15) × 10^–4^	(6.82 ± 0.98) × 10^–12^
LS12-1	LS12	(3.49 ± 0.65) × 10^7^	(3.53 ± 1.22) × 10^–4^	(1.05 ± 0.46) × 10^–11^
CS2		(4.96 ± 3.48) × 10^7^	(3.26 ± 1.58) × 10^–4^	(7.24 ± 2.29) × 10^–12^

*k*_on_, *k*_off_, *K*_D_ values are means and standard deviations of at least two independent experiments.

### Selectivity and mutational analysis of CS1 and CS2 RNAs

**Figure 6.**
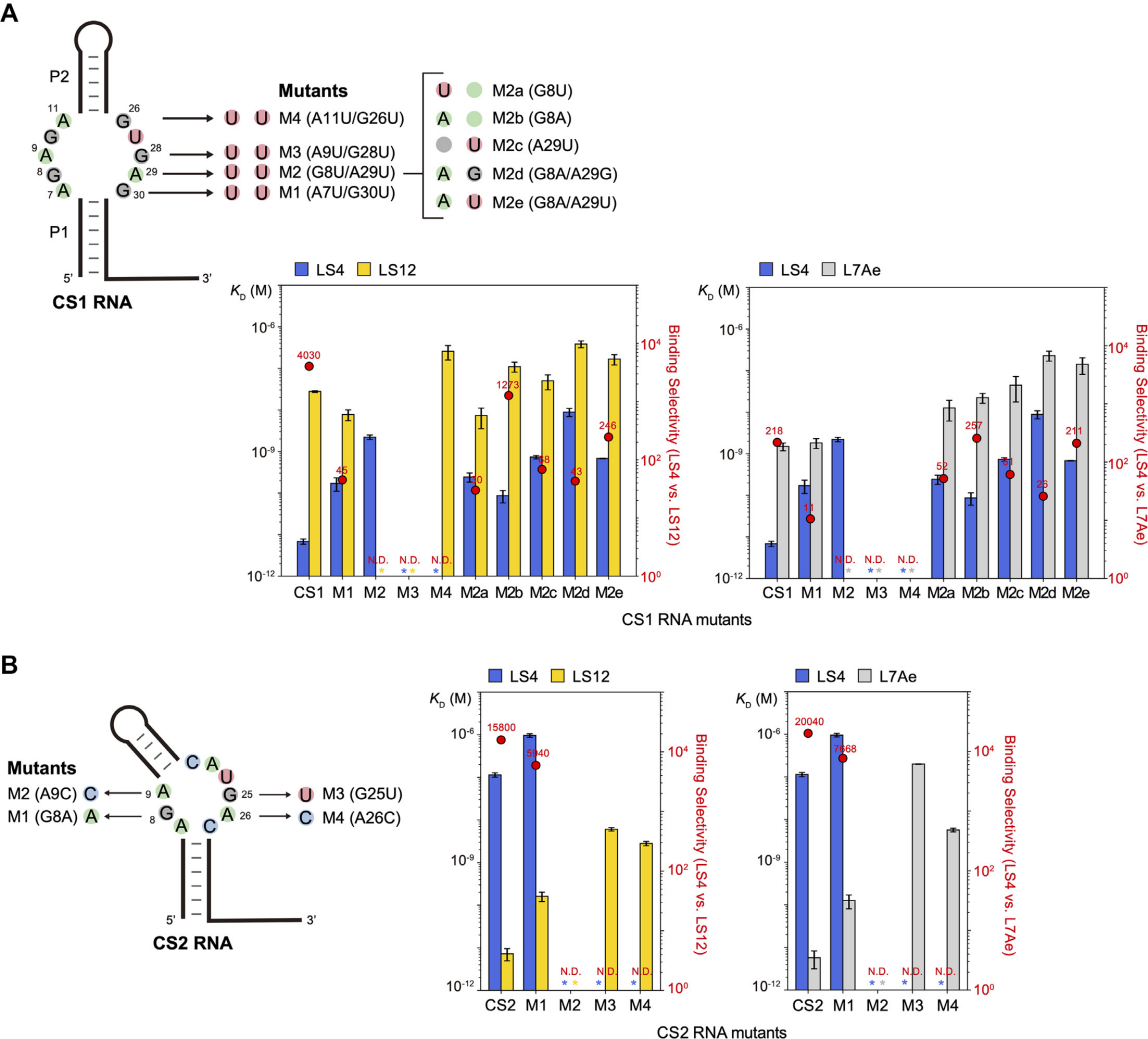
Binding selectivities of LS4, LS12, and L7Ae for (**A**) CS1 RNA and its mutants, and (**B**) CS2 RNA and its mutants. The bar graphs represent means and standard deviations of the *K*_D_ values derived from at least two independent experiments. Red dots represent binding selectivity (ratio of the *K*_D_ values) of the RNA for the two proteins. Asterisks indicate either no binding or weak binding with undetermined *K*_D_ value based on SPR. Binding selectivity could not be determined for these RNAs (N.D.). SPR sensorgrams and binding parameters are shown in Supplementary Figure S10 and Supplementary Table S4.

Binding selectivity of CS1 RNA was evaluated by SPR. The dissociation constant of CS1 RNA and LS12 was 2.75 × 10^–8^ M, and that of CS1 RNA and L7Ae was 1.49 × 10^–9^ M (Supplementary Figure S10 and Supplementary Table S4). Therefore, CS1 RNA selectively binds LS4 over LS12 and L7Ae by 4030-fold and 218-fold, respectively (Figure 6A). The measured binding affinities of CS1 RNA with the RBPs are consistent with the observed enrichment factors in the re-selection experiment (Figure 4B).

It is known that G•A mismatches in the core of Kt RNAs form *trans* G (sugar)•A (Hoogsteen) type base pairs (55). We found four putative G•A base pairs (A7/G30, G8/A29, A9/G28, and A11/G26) that may exist in the symmetric internal loop of CS1 RNA (Figure 6A). To examine the contribution of the putative G•A base pairs to the affinity with LS4, we systematically mutated each G•A pair to a U.U mismatch (M1: A7U/G30U, M2: G8U/A29U, M3: A9U/G28U, M4: A11U/G26U). Interestingly, as the mutation gets closer to the P2 stem, the binding affinity clearly decreased (CS1-M1 and CS1-M2) and no binding was observed with CS1-M3 and CS1-M4. This suggests that G•A pairs close to the P2 stem (i.e., A9/G28 and A11/G26) play an important role in binding. Among these four mutants, CS1-M2 retained nanomolar affinity (*K*_D_ = 2.21 × 10^–9^ M) for LS4 but its affinity for LS12 and L7Ae was not measurable (*K*_D_ > ∼1 μM). This prompted us to characterize additional mutants at these positions (M2a: G8U, M2b: G8A, M2c: A29U, M2d: G8A/A29G, M2e: G8A/A29U). CS1-M2b RNA exhibited respectable binding selectivity for LS4 (1273-fold over LS12) with a lower *K*_D_ (8.65 × 10^–11^ M). The possibility of tuning the binding affinity while preserving selectivity by mutating CS1 RNA may be advantageous for adapting the RNA–RBP pair for specific applications.

We next examined the binding affinities of CS2 RNA with LS4 and L7Ae. CS2 RNA bound LS4 at 1.14 × 10^–7^ M *K*_D_ which is 15800-fold greater than that for LS12 (7.24 × 10^–12^ M). On the other hand, the *K*_D_ value of CS2 RNA and L7Ae was 5.71 × 10^–12^ M (Supplementary Figure S10 and Supplementary Table S4) which was comparable to that of CS2 RNA and LS12 (Figure 5B). Therefore, although CS2 RNA cannot discriminate between LS12 and L7Ae, CS2 RNA selectively binds L7Ae 20 000-fold more strongly compared to LS4. These binding affinities were also consistent with the RNA binding trends in the re-selection experiment (Figure 4C).

We introduced several single-base mutations in the putative G•A pairs (G8/A26 and A9/G25) in the asymmetric internal loop of CS2 RNA (M1: G8A, M2: A9C, M3: G25U, M4: A26C) (Figure 6B). LS12 and L7Ae both showed similar degree of increase in *K*_D_ except for M3. Impact of the M3 mutation on binding affinity was more pronounced for L7Ae (*K*_D_ = 2.00 × 10^–7^ M) than for LS12 (*K*_D_ = 6.02 × 10^–9^ M) (Supplementary Figure S10 and Supplementary Table S4). This observation may be explained by the E34S substitution in LS12 (Figure 3C). It was shown that E34 in L7Ae contacts guanine in the G•A pair adjacent to the bulge in Kt-7 RNA (54) which corresponds to G25 in CS2 that was mutated to a uridine in CS2-M3 RNA.

The affinities of LS4 and LS12 for the box C/D Kt RNA were significantly impaired (*K*_D_ = 5.99 × 10^–7^ M and 1.45 × 10^–8^ M, respectively) compared to the box C/D Kt – L7Ae interaction (*K*_D_ = 2.13 × 10^–11^ M). (Supplementary Figure S10 and Supplementary Table S4). Therefore, these synthetic RBPs exhibit high selectivity for the cognate synthetic RNAs over the native L7Ae binding RNA motif. Overall, LS4 and CS1 RNA, as well as LS12 and CS2 RNA, were shown to be highly selective and orthogonal RNA–RBP pairs with picomolar affinities (Figure 6A and B).

## DISCUSSION

We used SPR to measure the L7Ae binding to box C/D Kt RNA resulting in a *K*_D_ value of 2.13 × 10^–11^ M (Figure 2B and Table 1) which is consistent with the result by the Lilley group who reported 10 pM *K*_D_ for another Kt motif (Kt-7) based on Förster resonance energy transfer (FRET) measurement using a stopped-flow mixer (55). However, Inoue and coworkers have reported significantly higher *K*_D_ values for box C/D RNA measured by SPR (between 1.6 and 2.5 nM) (13,32,42). We attribute the discrepancy to several possible factors. In two of the three studies (13,42), the researchers immobilized L7Ae protein on an SPR chip via EDC/NHS chemistry, and RNA was injected as the analyte. Immobilization of protein via primary amine groups results in heterogenous orientations of the immobilized protein some of which may negatively affect binding. In fact, some lysine residues in L7Ae are involved in RNA binding (54,63). Moreover, the negatively charged carboxymethylated dextran-functionalized sensor chip surface can reduce the local RNA analyte concentration due to electrostatic repulsion (64). The RNA was immobilized and L7Ae was injected as the analyte in another study that reported 1.6 nM *K*_D_ (32). While this experimental setup was similar to that of our study, we speculate that the recombinant L7Ae preparation may have caused the different observations. Turner and Lilley have pointed out that RNA contamination due to cellular RNAs copurified with L7Ae can affect affinity measurement, and they included a heparin chromatography step to minimize such interference (55). In our case, we treated the *E. coli* crude extracts containing recombinant proteins with ribonuclease A and deoxyribonuclease I to reduce nucleic acid contamination, and the proteins were affinity purified using two separate tags.

Hara *et al.* have reported their efforts to engineer L7Ae as a RBP scaffold to recognize non-natural RNA (13). They started with L7KK, a double mutant of L7Ae (K37A/K79A), which had a ∼51-fold reduced affinity for box C/D RNA and performed SELEX to isolate RNA motifs that bind to L7KK. While they discovered a compact aptamer H23 that recognizes L7KK, H23 also bound L7Ae with a comparable affinity. Consequently, they were not able to discover an orthogonal RNA–RBP pair, highlighting the challenges in engineering orthogonal RNA–RBP pairs by a single-library strategy. In PD-SELEX, it was anticipated that the selected RNA–RBP pairs would be orthogonal to the parental pair (box C/D Kt and L7Ae). Indeed, both LS4 and LS12 showed reduced affinity to box C/D Kt. However, while CS1 RNA showed 218-fold selectivity for LS4 over L7Ae, CS2 RNA bound LS12 and L7Ae with comparable affinity. In retrospect, inclusion of a negative selection step could have improved the chances of discovering other orthogonal RNA–RBP pairs. This could have been achieved by including excess L7Ae protein as a competitor during the RNA-phage binding step.

Since the first application of L7Ae and box C/D Kt RNA for translational regulation in mammalian cells by Saito *et al.* (32), the RNA–RBP pair has been a popular module for applications in synthetic biology. However, Randau *et al.* reported that overproduction of L7Ae causes cytotoxicity in *E. coli* suggesting that L7Ae binds to endogenous RNAs in *E. coli* (36). It has also been reported that overexpression of L7Ae in mammalian cells causes slower growth and/or cell death (41,65). This is probably because archaeal L7Ae can bind to broad Kt structures which are conserved in many species from archaea to humans (53,57). The evolved LS4 protein strongly binds CS1 RNA with a *K*_D_ value of 6.82 × 10^–12^ M (Figure 5 and Table 2) while its affinity for the box C/D Kt and another putative Kt RNA (CS2 RNA) is significantly compromised (*K*_D_ > 100 nM) (Supplementary Figure S10 and Supplementary Table S4). Moreover, we confirmed that without nuclease treatment, *E. coli* expressed L7Ae and LS12 are copurified with abundant cellular DNA and RNA in cell lysate, consistent with the observations by Turner and Lilley (55), suggesting nonspecific interactions with nucleic acids. However, LS4 was copurified with a markedly lower amount of nucleic acids under the same conditions (Supplementary Figure S11). While it remains to be experimentally tested, LS4 may exhibit reduced cytotoxicity for synthetic biology applications compared to L7Ae.

To date, in vitro and *E. coli* or yeast cell-based in vivo library-vs-library selections have been reported to identify protein-protein (21–25), protein-peptide (26), peptide-peptide (27,28), and small molecule-protein (29,30), small molecule-RNA (31) pairs. This study is the first library-vs-library selection for RNA-protein interactions. In cell-based library-vs-library selections (22,25–28), two different genes are transformed into the cell, and binding between the two components results in expression of a drug resistance gene or a reporter gene which is used to enrich the interacting partners. However, combinations of the binding partners that can be explored using cell-based methods are limited by the transformation efficiency, or ∼10^9^ pairs (66). The sequence space that can be explored by in vitro library-vs-library selections significantly exceeds those that can be probed by cell-based experiments. For example, Lerner and coworkers carried out library-vs-library selection using yeast-displayed single-chain antibody fragment library with a diversity of 2 × 10^7^ and phage-displayed antigen library with a diversity of ∼10^7^, and successfully enriched an antibody-antigen pair from >10^14^ possible combinations (21). Our PD-SELEX experiment started with up to 4.4 × 10^20^ possible RNA–RBP combinations, significantly higher than previous library-vs-library selection experiments. However, a caveat with highly complex library-vs-library selection is the low concentrations of the individual binding partners in solution. It can be imagined that if the binding affinity between an RNA motif and a protein mutant is weak, such a pair may not be able to survive the first round of selection due to the low concentration of the bound pair. Here, the relevant concentration is not necessarily the absolute concentration of a single RNA or protein sequence but could be the total effective concentration of multiple sequences that contain a consensus motif. Our PD-SELEX yielded RNA–RBP pairs with ∼7 pM *K*_D_. This strong affinity combined with the relatively small RNA motifs (CS1 and CS2) that are represented more frequently in the randomized RNA library (relative to larger and more complex RNA motifs) may have contributed to the successful enrichment the RNA–RBP pairs. In principle, RNA–RBP pairs with lower affinity (e.g. nanomolar *K*_D_) should also be selectable by PD-SELEX. In Bowley *et al.*, an antigen-antibody pair with a *K*_D_ of 50 nM was selected from phage-displayed antibody and yeast-displayed antigen libraries (21). However, it may be necessary to adjust the library complexity and/or the selection conditions to optimize the enrichment efficiency for the expected or desired affinity range. This can be achieved by performing a mock selection experiment as shown in Figure 2.

In conclusion, we designed and executed PD-SELEX to select orthogonal RNA–RBP pairs from RNA and phage-displayed RBP libraries. L7Ae mutants LS4 and LS12 and their cognate RNA binding motifs (CS1 and CS2 RNAs) show low picomolar affinities which are among the strongest RNA-protein interactions observed in natural or synthetic systems (67–70). We expect PD-SELEX to be a useful and efficient strategy for engineering and analysis of RNA-protein interactions.

## DATA AVAILABILITY

All data and materials are available upon request.

## Supplementary Material

gkab527_Supplemental_File
