# Supplementary material for: Directed evolution of orthogonal RNA–RBP pairs through library-vs-library in vitro selection
Source: Nucleic Acids Res. 2021 Jul 5;50(2):601–16. doi: 10.1093/nar/gkab527 (PMC8789040; doi:10.1093/nar/gkab527)
Supplement: gkab527_Supplemental_File [file gkab527_supplemental_file.docx]

**SUPPLEMENTARY DATA**

Directed evolution of orthogonal RNA-RBP pairs through libary-vs-library in vitro selection

Keisuke Fukunaga and Yohei Yokobayashi*

*Nucleic Acid Chemistry and Engineering Unit, Okinawa Institute of Science and Technology Graduate University, Onna, Okinawa 904 0495, Japan*

*E-mail: yohei.yokobayashi@oist.jp

|  | Page |
| --- | --- |
| **Supplementary Methods** | S2 |
| **Supplementary Figures S1-11 and Tables S1-4**   - Figure S1. Plasmid map of bacterial expression vector - Figure S2. SDS-PAGE of recombinant proteins used for SPR and EMSA - Figure S3. DNA library construction - Figure S4. Amino acid sequences of phage-displayed L7Ae and dL7Ae and Western blotting - Figure S5. Amino acid bias of initial PD pool - Figure S6. Western blotting of recombinant proteins used for re-selection experiment - Figure S7. Control experiment for Figure 2E - Figure S8. Sequence trends of archaeal L7Ae and laboratory-evolved proteins - Figure S9. EMSA - Figure S10. SPR sensorgrams - Figure S11. His-tag pulldown of recombinant proteins without nuclease treatment - Table S1. In vitro transcribed RNAs - Table S2. Oligo DNAs used in this study - Table S3. Detailed summary of selection parameters - Table S4. RNA-RBP binding properties determined by SPR | S3  S4  S5  S6  S7  S8  S9  S10  S11  S12  S13  S14 |
| **Supplementary References** | S15 |

**SUPPLEMENTARY METHODS**

**Western blot analysis**

Proteins were resolved by SDS-PAGE (4-15% TGX gel or Any kD TGX gel, Bio-Rad) and transferred onto a polyvinylidene difuoride membrane (Immun-Blot PDVF Membrane, Bio-Rad) using a semidry blotter (Trans-Blot Semi-Dry Electrophoretic Transfer Cell, Bio-Rad). After blocking with Blocking One (Nacalai), the blot was incubated with horseradish peroxidase-conjugated Strep-Tactin (1:10000 dilution, Bio-Rad). After several washing with 1× tTBS (Nacalai), the blot was incubated with Western BLoT Hyper HRP Substrate (TaKaRa). Luminescence was detected using LuminoGraph II (ATTO).

**Electrophoretic mobility shift assay (EMSA)**

Cy5-labeled RNAs (HPLC purification grade) were purchased from FASMAC: Cy5-CS1 RNA (5’-/Cy5/ AGAGGCAGAGAAAGGGAAACCUUGUGAGGCCUCU-3’), Cy5-CS2 RNA (5’-/Cy5/AGAGGCAGAGAAGGA AACUUCCAUGACGCCUCU-3’). Cy5-labeled RNA (5 nM, 1 µL) was mixed with 1 µL of 5× EMSA binding buffer (50 mM HEPES-KOH, pH 7.5, 700 mM KCl, 50 mM NaCl, 5 mM MgCl_2_, 0.5 mM TCEP-Na, 0.05% v/v Tween-20, 50 μg/mL yeast tRNA), and 2 µL of ultrapure water was added. Various concentrations of protein solutions were prepared with 1× EMSA binding buffer, and 5 µL of protein solution was mixed with 4 µL of the RNA solution. After adding 1 µL of 10× EMSA loading buffer (10 mM HEPES-KOH, pH 7.5, 40% w/v sucrose), the mixture was incubated overnight (> 16 h) at 4 °C in the dark. It should be noted that long incubation time was allowed to ensure that the system reaches equilibrium due to the slow dissociation rate ([1](#_ENREF_1)). The samples (10 µL) were resolved by 8% native PAGE using 0.5× TBE buffer (44.5 mM Tris-borate, pH 8.3, 1 mM EDTA-Na) as a running buffer. Electrophoresis was carried out for 30 min at constant voltage of 200 V at room temperature, and the gel was imaged with Typhoon FLA9500 (GE Healthcare). Cy5 was excited with 635 nm laser and detected through LPR (665 nm long pass) filter. The voltage for photomultiplier tube (PMT) was set to 800. Quantification was performed using Image J software ([2](#_ENREF_2)). Experiments were repeated twice to ensure reproducibility. Plots were generated using GraphPad Prism 6 software.

**His-tag pulldown of recombinant proteins without nuclease treatment**

*E. coli* BL21 (DE3) cells carrying an RBP expression plasmid or pRARE plasmid (Novagen) were pre-cultured overnight in LB medium supplemented with 50 µg/mL carbenicillin and 10 µg/mL chloramphenicol at 30 °C and diluted 20-fold with fresh 2 mL LB medium supplemented with the same antibiotics. After 2 h culture at 30 °C, IPTG was added to a final concentration of 0.4 mM, and the cells were further cultured overnight (> 16 h) at 30 °C. The cells were harvested by centrifugation at 8000 × *g* for 5 min at 4 °C and then resuspended in 0.5 mL lysis buffer (50 mM phosphate-Na, pH 7.7, 300 mM NaCl, 1 mM 2-mercaptoethanol, 5 mM imidazole, 0.5% v/v Triton X-100, 0.1 mg/mL lysozyme). The cells were disrupted by ultrasonication, and the crude cell extract was cleared by centrifugation at 15000 × *g* for 5 min at 4 °C. The supernatant was transferred to a new tube and incubated with 25 µL of Dynabeads TALON (Invitrogen) at room temperature for 30 min. The magnetic beads were washed twice with 500 µL lysis buffer and twice with 500 µL His-tag purification buffer C (50 mM phosphate-Na, pH 7.7, 300 mM NaCl, 5 mM imidazole, 0.01% v/v polyoxyethylene (23) lauryl ether). His-tagged proteins were eluted with 50 µL His-tag elution buffer 2 (50 mM phosphate-Na, pH 7.7, 300 mM NaCl, 150 mM imidazole, 0.01% v/v polyoxyethylene (23) lauryl ether).

**SUPPLEMENTARY FIGURES AND TABLES**

**Figure S1.** Plasmid map of the bacterial expression vector. The recombinant proteins were tagged with an N-terminal Twin-Strep-tag and a C-terminal 6× His-tag. The map was generated by Benchling (https://benchling.com/). Amino acid sequences of the recombinant proteins are shown in Figure S6.


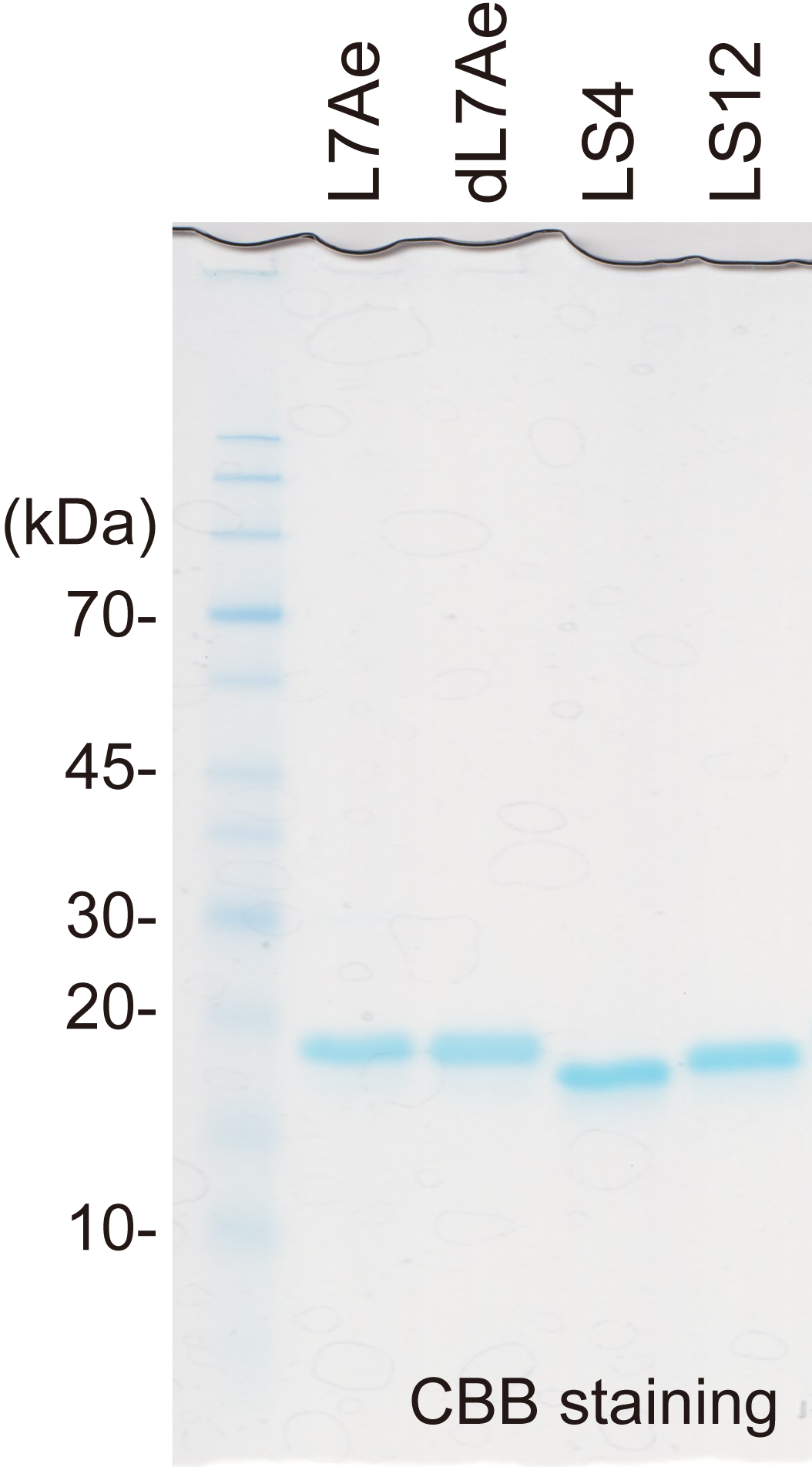


**Figure S2 (related to Figure 2B, 5, 6, S9 and S10).** SDS-PAGE of recombinant proteins used for SPR and EMSA analyses. Imaging of CBB-stained gel was performed on CanoScan LiDE 400 (Canon). Calculated molecular weights according to ProtParam ([3](#_ENREF_3)) are L7Ae: 19.3 kDa, dL7Ae: 19.0 kDa, LS4: 19.3 kDa, LS12: 19.2 kDa. Minor differences in the SDS-PAGE mobilities are presumably due to amino acid substitutions ([4](#_ENREF_4)).


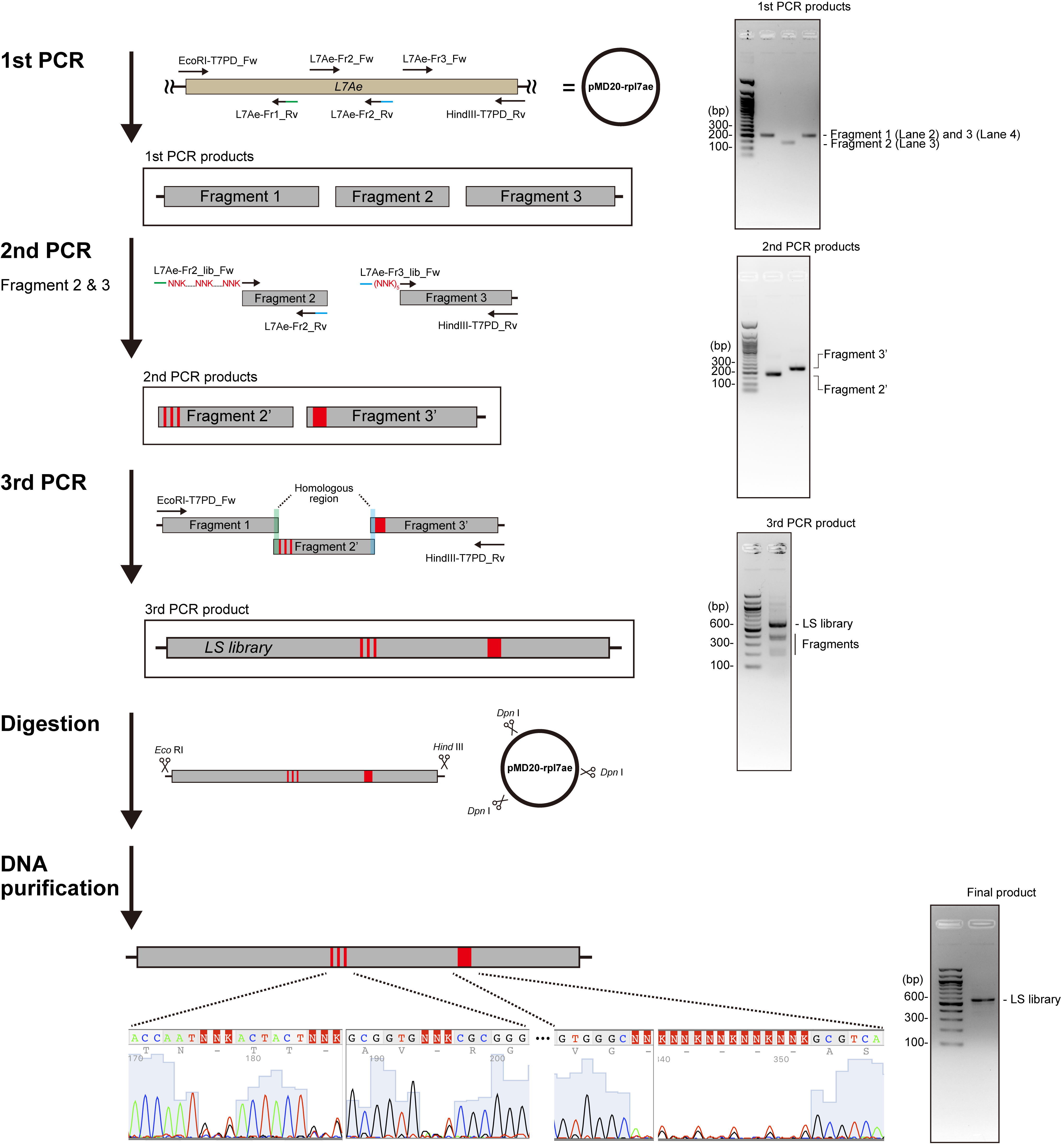


**Sequence of DNA library**

-GAATTCTGGTGGCGGTGGTTCTGACTATAAGGATCACGACGGTGATTACAAAGATCACGACATCGATTACAAGGACGACGACGACAAGGGTGGCGGAGGATCGTACGTCAGATTCGAAGTTCCCGAAGATATGCAAAATGAAGCTCTTTCCCTTCTTGAGAAGGTCCGGGAGAGCGGTAAGGTCAAGAAAGGTACCAATNNKACTACTNNKGCGGTGNNKCGCGGGTTGGCAAAGCTGGTATATATCGCCGAAGATGTGGACCCGCCGGAAATCGTAGCTCATCTCCCACTTCTTTGCGAAGAGAAAAATGTTCCATATATCTACGTTAAATCAAAGAATGATCTGGGCCGAGCCGTGGGCNNKNNKNNKNNKNNKGCGTCAGCCGCTATAATAAATGAAGGGGAGCTTAGAAAGGAGCTTGGTAGTCTCGTGGAGAAGATCAAAGGACTGCAAAAACGCTCCCACATGCACCTGGAAGGCGGTGGGGGAAGTGGGGGTGGAGGCTCTGGAGGAGGGGGGTCCGCTTCCTGGAGCCATCCCCAATTTGAGAAAGGCGCTTAATAAGCTT-

**Sequence of protein library**

-NSGGGGSDYKDHDGDYKDHDIDYKDDDDKGGGGSYVRFEVPEDMQNEALSLLEKVRESGKVKKGTNXTTXAVXRGLAKLVYIAEDVDPPEIVAHLPLLCEEKNVPYIYVKSKNDLGRAVGXXXXXASAAIINEGELRKELGSLVEKIKGLQKRSHMHLEGGGGSGGGGSGGGGSASWSHPQFEKGA

**Figure S3 (related to Figure 3A).** Construction of the DNA fragment encoding the L7Ae mutants for PD library (see Construction of PD library, Material and Methods in the main text). L7Ae and Strep-tag II are shown in aqua blue and orange, respectively. Randomized positions are shown in red. N = A, C, G or T; K = G or T; X = any of the 20 genetically coded amino acids. Restriction enzyme recognition sites (*Eco* RI and *Hind* III) are underlined. The Sanger sequencing chromatogram was prepared using 4Peaks software (Griekspoor, A. and Groothuis, T., nucleobytes.com).

**
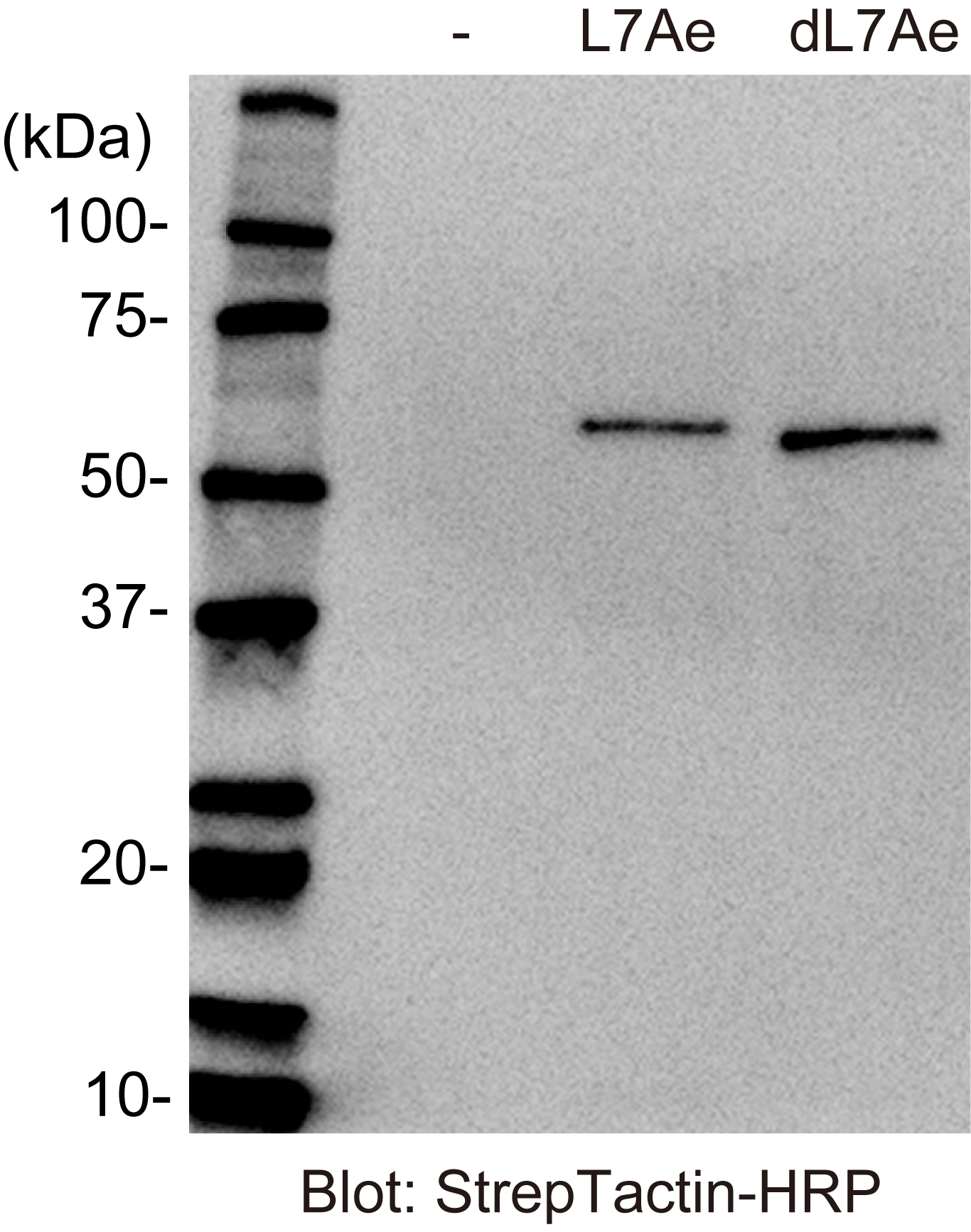
Sequence of phage displayed L7Ae**

Gp10B-GGGGSDYKDHDGDYKDHDIDYKDDDDKGGGGSYVRFEVPEDMQNEALSLLEKVRESGKVKKGTNETTKAVERGLAKLVYIAEDVDPPEIVAHLPLLCEEKNVPYIYVKSKNDLGRAVGIEVPCASAAIINEGELRKELGSLVEKIKGLQKRSHMHLEGGGGSGGGGSGGGGSASWSHPQFEKGA

**Sequence of phage displayed dL7Ae**

Gp10B-GGGGSEGGVAMPGAEDDVVGGGGSYVRFEVPEDMQNEALSLLEKVRESGKVKKGTNKTTEAVEEGLAKLVYIAEDVDPPEIVAHLPLLCEEKNVPYIYVKSKNDLGRAVGGGGGSASAAIINEGELRKELGSLVEKIKGLQKRSHMHLEGGGGSGGGGSGGGGSASWSHPQFEKGA

**Figure S4 (related to Figure 2C).** Amino acid sequences of the phage displayed L7Ae and dL7Ae, and their expression confirmed by Western blotting. Calculated molecular sizes according to ProtParam ([3](#_ENREF_3)), Gp10B-L7Ae: 59.6 kDa, Gp10B-dL7Ae: 55.2 kDa. Gp10B is the minor coat protein of bacteriophage T7 ([5](#_ENREF_5)). L7Ae and Strep-tag II sequences are shown in aqua blue and orange, respectively. Substituted residues are highlighted in yellow.


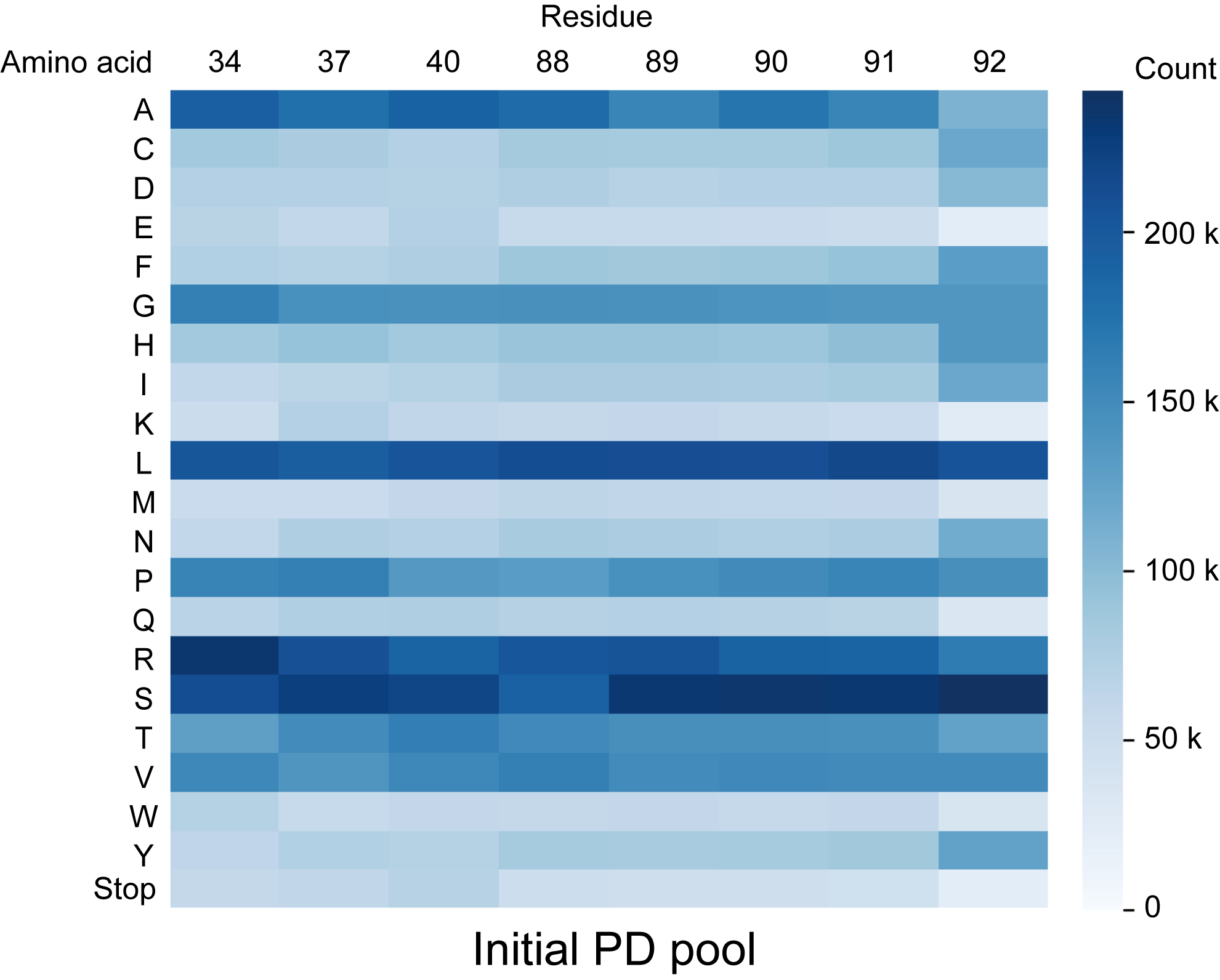


**Figure S5.** Amino acid frequencies of the randomized residues in the initial PD library analyzed by Illumina sequencing. The observed amino acid bias reflects the NNK degenerate codon which statistically favors some amino acids over others.

**Sequence of recombinant proteins**

MGSAWSHPQFEKGGGSGGGSGGSAWSHPQFEKGGGGSYVRFEVPEDMQNEALSLLEKVRESGKVKKGTNXTTXAVXRGLAKLVYIAEDVDPPEIVAHLPLLCEEKNVPYIYVKSKNDLGRAVGXXXXXASAAIINEGELRKELGSLVEKIKGLQKRSHMHLEGGGGSGGGGSGGGGSHHHHHH


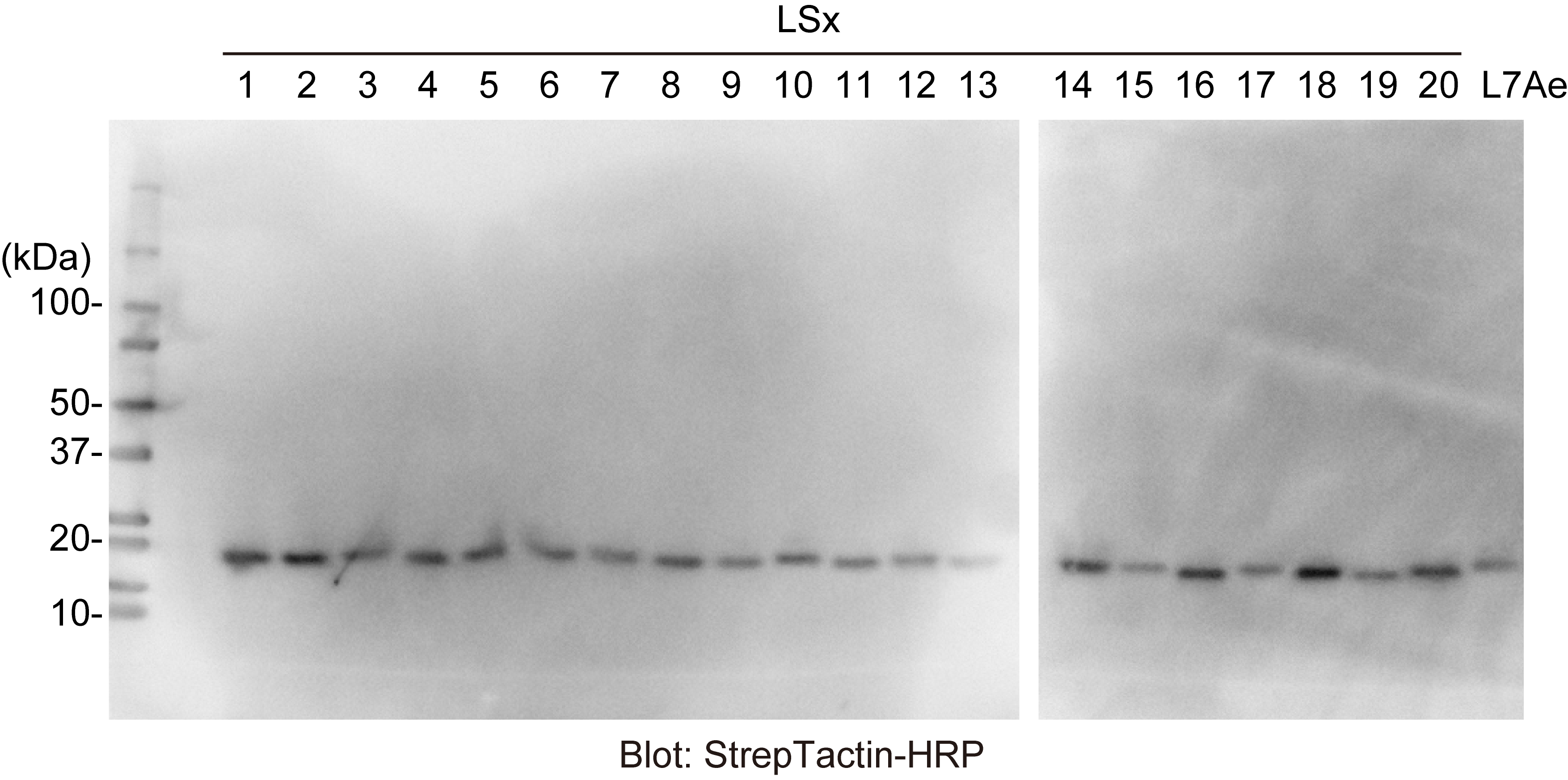


**Figure S6 (related to Figure 4).** Western blotting of recombinant proteins used for the re-selection experiment. Twin-Strep-tag, LS protein, and His-tag are shown in orange, aqua blue and green, respectively. Amino acid sequences X are shown in Figure 3c.


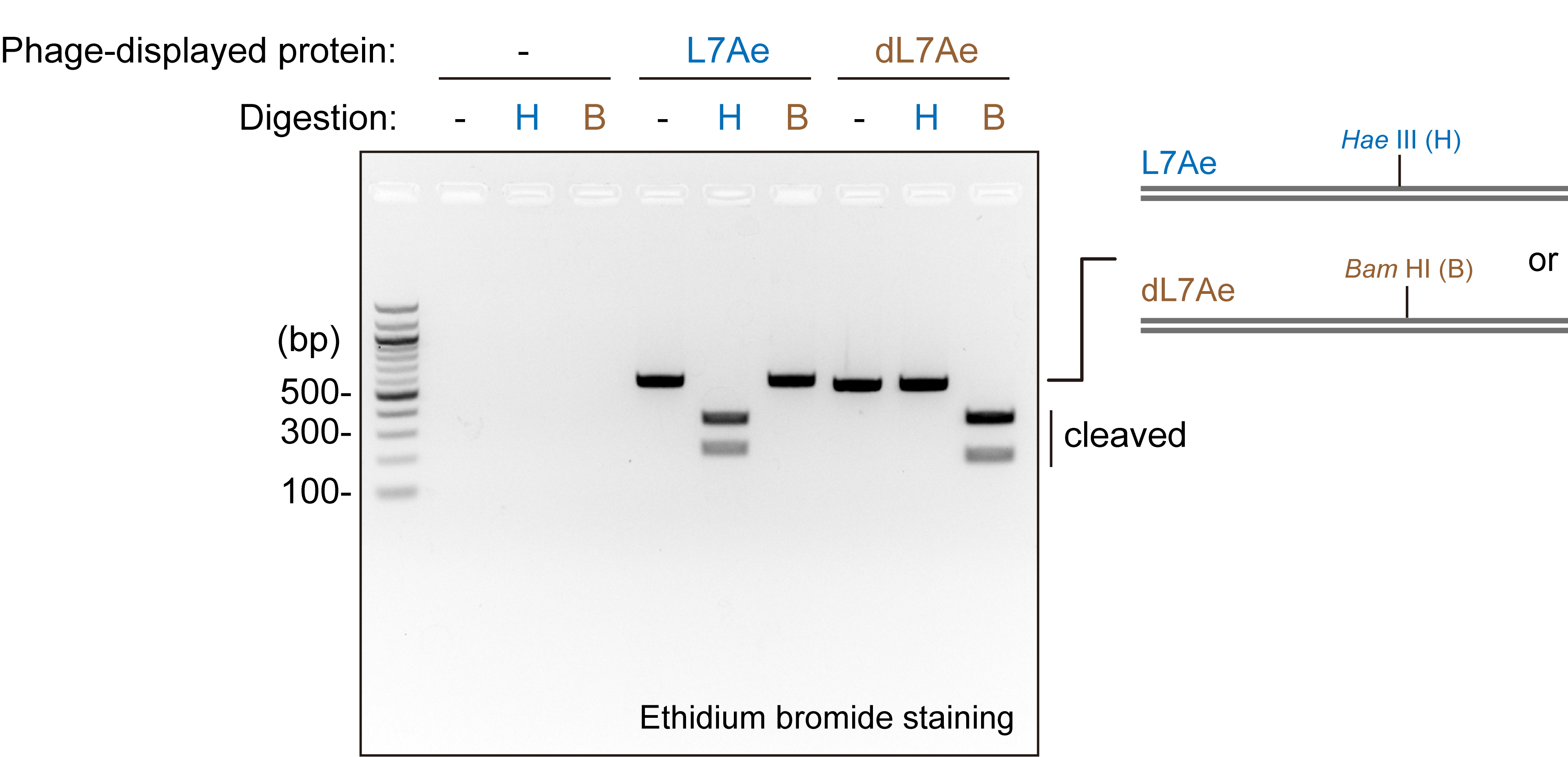


**Figure S7.** Control experiment for Figure 2E. PCR products were digested with *Hae* III (H) or *Bam* HI (B), and then separated by 2% agarose gel electrophoresis. Phages displaying no exogenous protein (mock phage particle) were utilized as a negative control for PCR. DNAs containing a restriction enzyme recognition site were specifically and completely digested by the corresponding restriction enzyme.

**
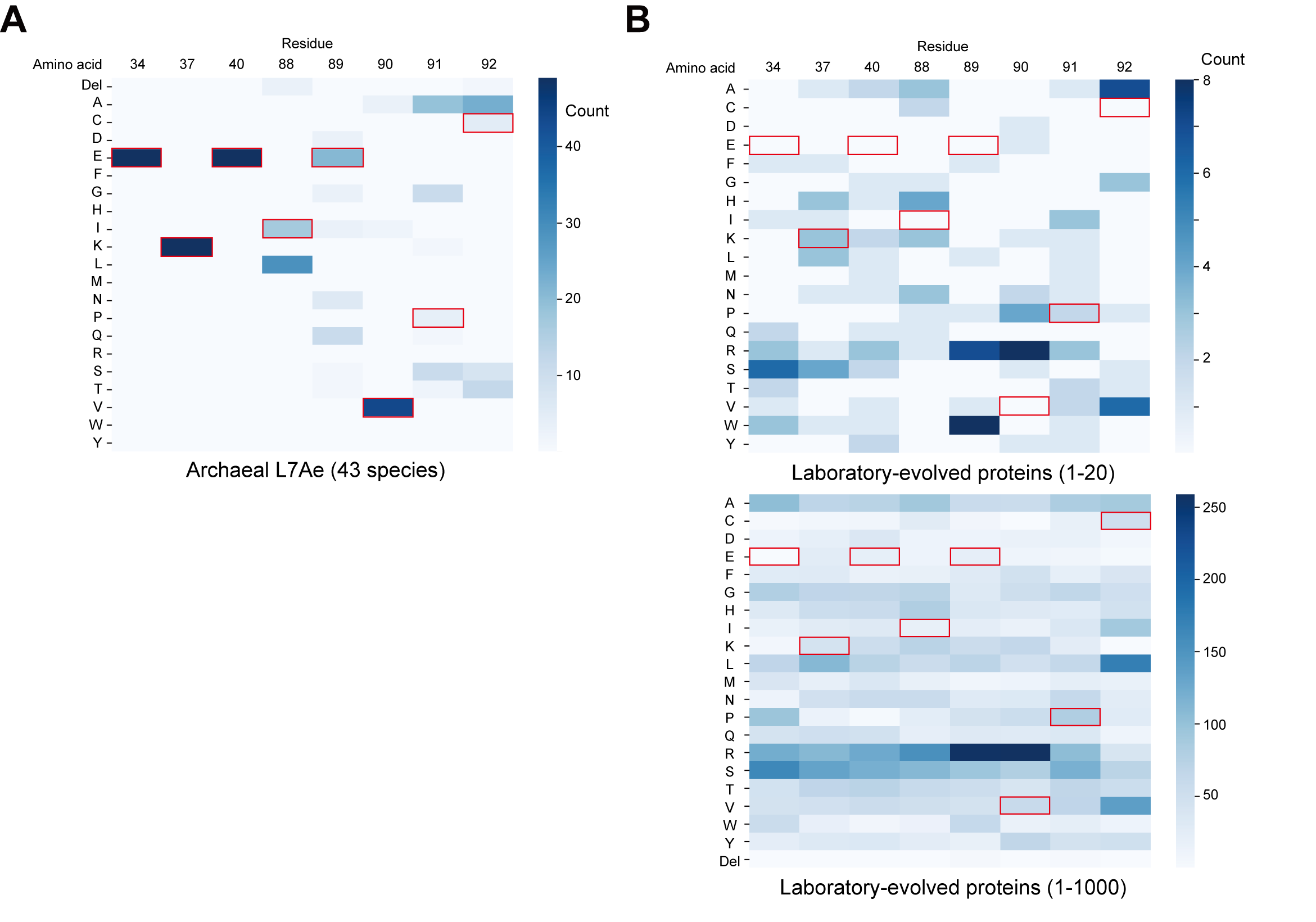
**

**Figure S8 (related to Figure 3C).** Frequencies of amino acids in the mutated residues in L7Ae in (A) the archaeal L7Ae variants, and (B) the laboratory-evolved proteins (upper panel: 20 most abundant sequences, lower panel: 1000 most abundant sequences). The amino acids of *A. fulgidus* L7Ae at these positions are shown in red boxes. Del: deletion. Protein sequences of archaeal L7Ae were retrieved from UniProt database ([6](#_ENREF_6)), and sequence alignment was performed using CLUSTALW ([7](#_ENREF_7)). The archaeal L7Ae proteins included in the analysis are (43 species, 49 strains) : *T. volcanium*, *T. acidophilum*, *P. torridus*, *M. maripaludis*, *M. aeolicus*, *M. jannaschii*, *S. islandicus* (strains L.S.2.15, Y.N.15.51, Y.G.57.14, M.16.4, M.16.27, and M.14.25), *S. solfataricus*, *S. acidocaldarius*, *S. tokodaii*, *M. sedula*, *H. marismortui*, *H. walsbyi*, *N. pharaonis*, *H. salinarum* (strains ATCC 700922 and ATCC 29341), *H. lacusprofundi*, *M. barkeri*, *M. acetivorans*, *M. mazei*, *M. arvoryzae*, *M. thermoacetophila*, *M. smithii*, *M. stadtmanae*, *M. thermautotrophicus*, *P. horikoshii*, *P. abyssi*, *P. furiosus*, *T. kodakarensis*, *T. gammatolerans*, *T. onnurineus*, *M. kandleri*, *S. marinus*, *D. amylolyticus*, *I. hospitalis*, *H. butylicus*, *A. pernix*, *N. equitans*, *P. aerophilum*, *P. calidifontis*, *P. neutrophilum*, *A. fulgidus*, *N. maritimus*, and *C. symbiosum*. Heatmaps were generated using Python.


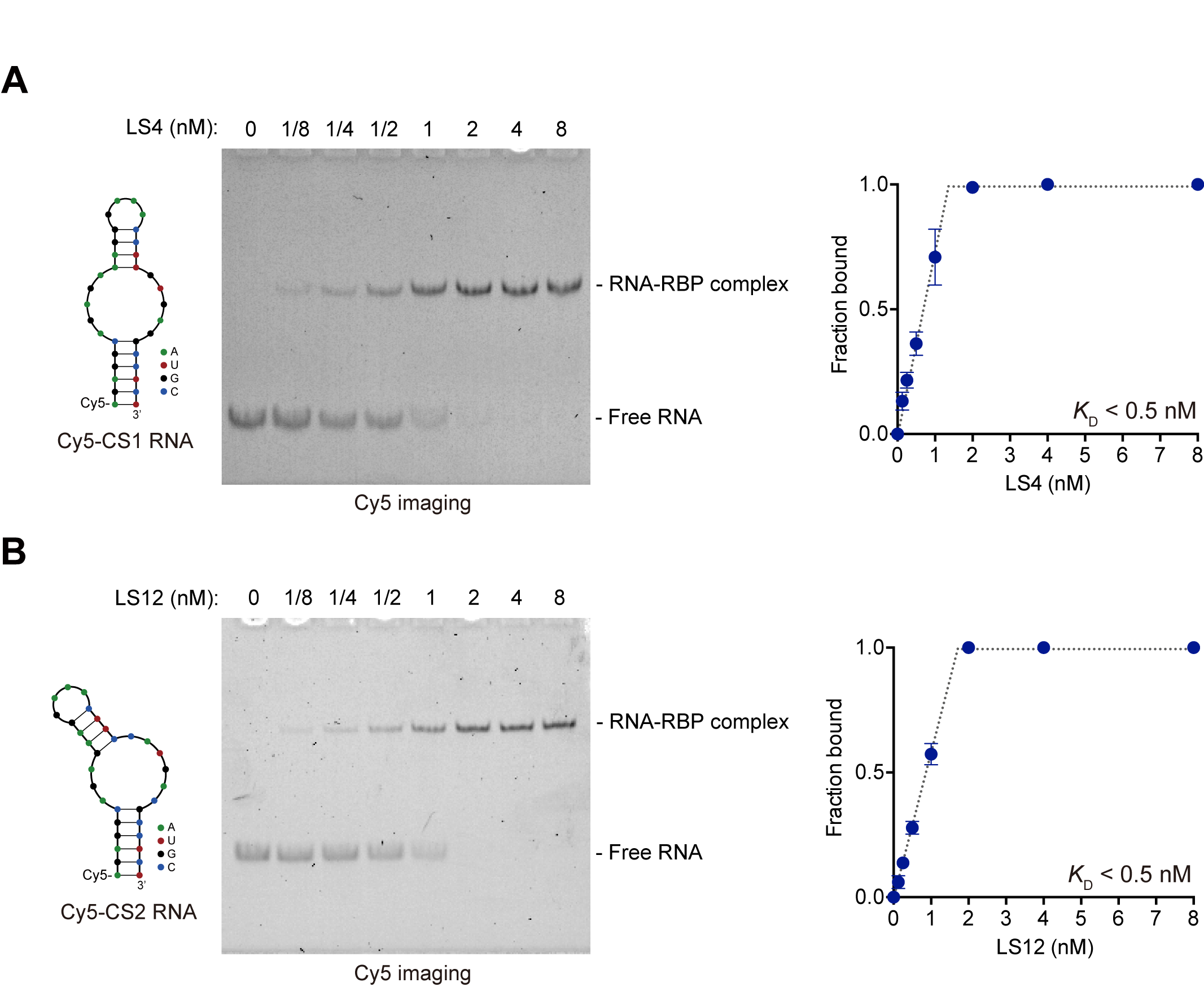


**Figure S9.** Electrophoretic mobility shift assay (EMSA) of (A) CS1 RNA – LS4 and (B) CS2 RNA – LS12 pairs. A recombinant protein (LS4 or LS12, 0-8 nM) was mixed with 0.5 nM 5’-Cy5-labeled RNA (CS1 or CS2) and separated by native PAGE. Secondary structures of the RNAs predicted by NUPACK are shown on the left side. It should be noted that there are minor sequence differences in the stem regions from the sequences of CS1 RNA and CS2 RNA used for SPR measurements in Figure 5. The graphs (right) show means and standard deviations of two independent experiments. The linear increase of the bound RNA fraction until saturation is indicative of the titration regime ([1](#_ENREF_1)). Therefore, it suggests that *K*_D_ is significantly smaller than the RNA concentration used (*K*_D_ << 0.5 nM).


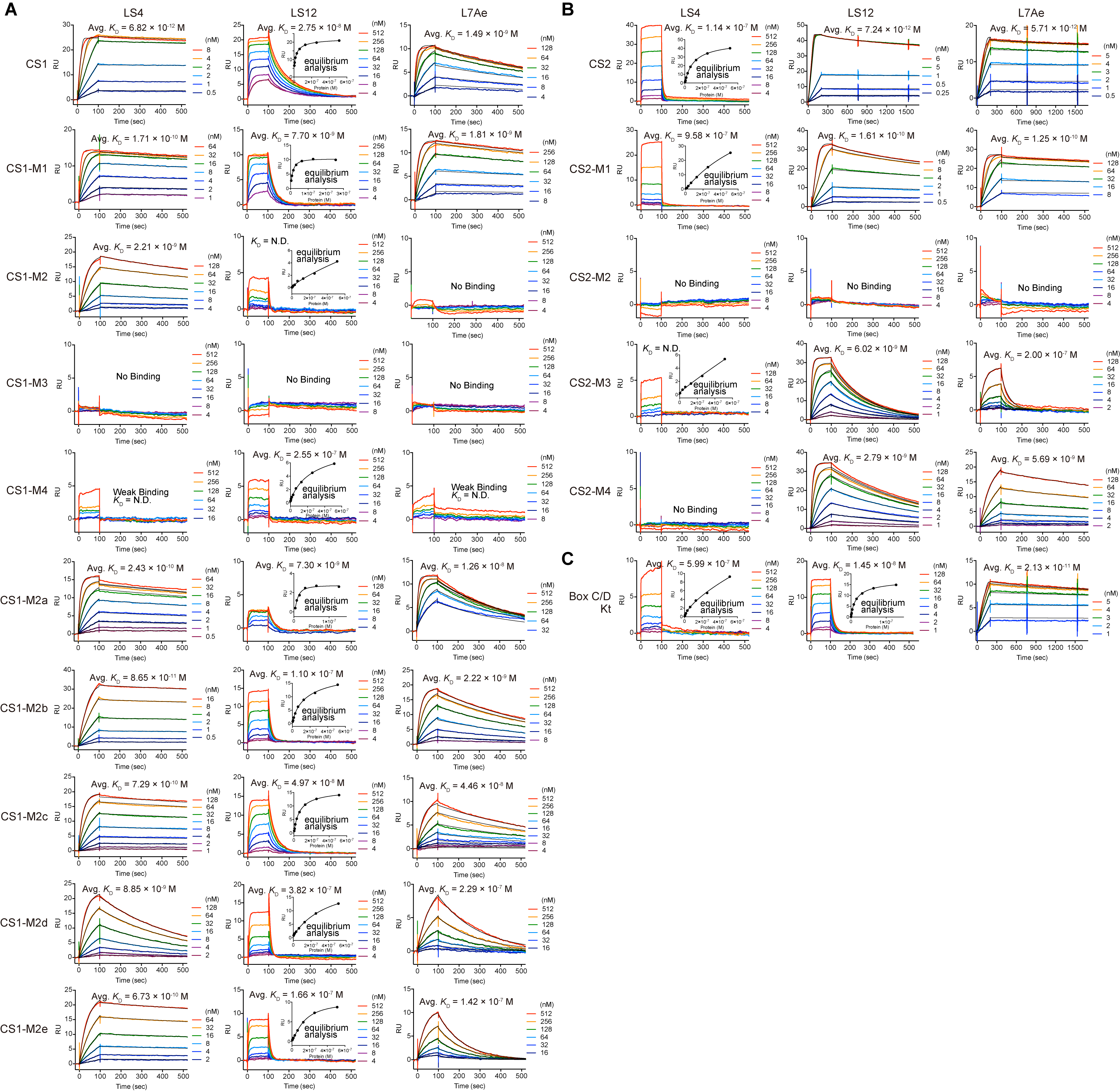


**Figure S10 (related to Figure 6).** Binding properties of LS4, LS12, and L7Ae for (A) CS1 RNA and its mutants, (B) CS2 RNA and its mutants, and (C) box C/D Kt RNA. Sensorgrams are shown in colored lines, and black lines represent curve fitting according to 1:1 binding model. RU stands for response unit. Two sensorgrams, “CS1 – LS4”, and “CS2 – LS12” are identical to those in Figure 5. The *K*_D_ values shown are means of at least two independent experiments.

**
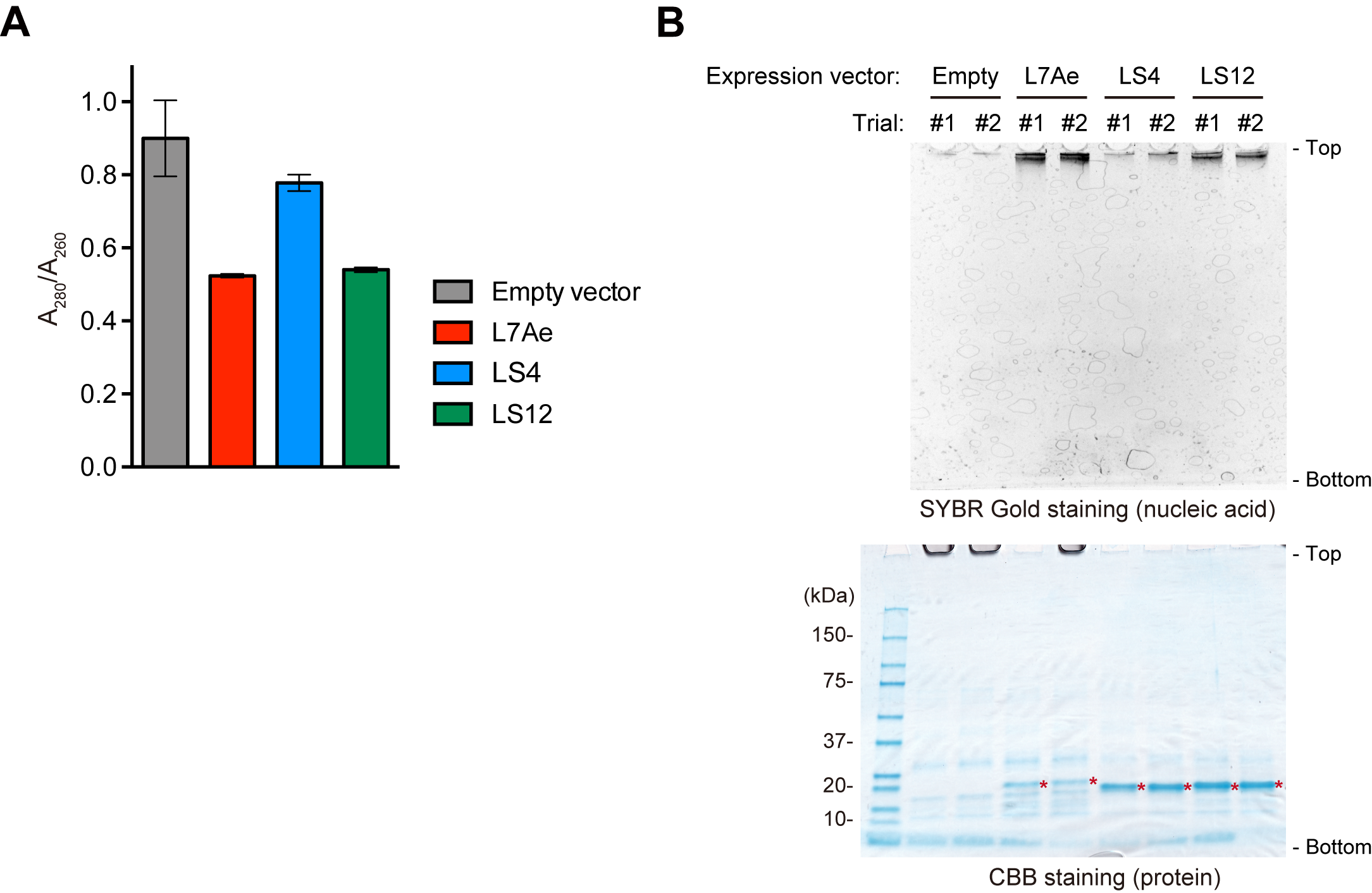
**

**Figure S11.** Copurification cellular nucleic acids with L7Ae, LS4, and LS12. (A) Absorbance ratio (A_280_/A_260_) indicates nucleic acid contamination in His-tag purified recombinant proteins without nuclease treatment. Bars and error bars represent mean and standard deviations of two experiments. The empty vector (pET-15b, Novagen) encodes a His-tagged peptide (MGSSHHHHHHSSGLVPRGSHMLEDPAANKARKEAELAAATAEQ, 4.6 kDa). Absorption of the purified samples in His-tag elution buffer was measured using NanoDrop One (Thermo Fisher Scientific). (B) His-tag purified peptide/proteins were separated by 8% denaturing PAGE (top) and 4-15% SDS-PAGE (bottom). Nucleic acid (> 500 nt) contaminations were observed in L7Ae and LS12 preparations while the LS4 preparation included lower amount of nucleic acids (top). The SDS-PAGE of the protein preparations shows full-length His-tagged proteins indicated by red asterisks (below).

**Table S1.** In vitro transcribed RNAs

| RNA | RNA sequence (5’-3’) |
| --- | --- |
| Kt_SPR | GGAUCGCGUGAUCCGAAAGGUGACGAUCCAAAUCGACGAAAGACACAGAACG |
| dKt_SPR | GGCUGGGGCGUCAUCCGAAAGGUGCCCCAGCCAAAUCGACGAAAGACACAGAACG |
| Kt_mock selection | GGCAGAAUCAUAAUGCUGGACUGGGCGUGAUGCGAAAGCUGACCCGGCCAUCGACGAAAGACACAGAACG |
| dKt_mock selection | GGCAGAAUCAUAAUGCUGGACUGGGGCGUCAUCCGAAAGGUGCCCCGGAUCCACAUCGACGAAAGACACAGAACG |
| N20L_library | GGCAGAGAAAGGCCAUACAAUCAUUGGNNNNNNNNNNNNNNNNNNNNCCAGUGGCAUCGACGAAAGACACAGAACG |
| LS4-1_Full | GGCAGAGAAAGGCCAUACAAUCAUUGGCCUUGUGAGGCCGUGUGUCUUCCAGUGGCAUCGACGAAAGACACAGAACG |
| CS1 | GGUGGCAGAGAAAGGCGAAAGCCUUGUGAGGCCAUCAAAUCGACGAAAGACACAGAACG |
| CS1-M1 | GGUGGCUGAGAAAGGCGAAAGCCUUGUGAUGCCAUCAAAUCGACGAAAGACACAGAACG |
| CS1-M2 | GGUGGCAUAGAAAGGCGAAAGCCUUGUGUGGCCAUCAAAUCGACGAAAGACACAGAACG |
| CS1-M3 | GGUGGCAGUGAAAGGCGAAAGCCUUGUUAGGCCAUCAAAUCGACGAAAGACACAGAACG |
| CS1-M4 | GGUGGCAGAGUAAGGCGAAAGCCUUUUGAGGCCAUCAAAUCGACGAAAGACACAGAACG |
| CS1-M2a | GGUGGCAUAGAAAGGCGAAAGCCUUGUGAGGCCAUCAAAUCGACGAAAGACACAGAACG |
| CS1-M2b | GGUGGCAAAGAAAGGCGAAAGCCUUGUGAGGCCAUCAAAUCGACGAAAGACACAGAACG |
| CS1-M2c | GGUGGCAGAGAAAGGCGAAAGCCUUGUGUGGCCAUCAAAUCGACGAAAGACACAGAACG |
| CS1-M2d | GGUGGCAAAGAAAGGCGAAAGCCUUGUGGGGCCAUCAAAUCGACGAAAGACACAGAACG |
| CS1-M2e | GGUGGCAAAGAAAGGCGAAAGCCUUGUGUGGCCAUCAAAUCGACGAAAGACACAGAACG |
| LS12-1_Full | GGCAGAGAAAGGCCAUACAAUCAUUGGCUUUUCCAUGACGCCAGUUCCAGUGGCAUCGACGAAAGACACAGAACG |
| CS2 | GGAUGCAGAGAACGAAAGUUCCAUGACGCAUCCAAAUCGACGAAAGACACAGAACG |
| CS2-M1 | GGAUGCAAAGAACGAAAGUUCCAUGACGCAUCCAAAUCGACGAAAGACACAGAACG |
| CS2-M2 | GGAUGCAGCGAACGAAAGUUCCAUGACGCAUCCAAAUCGACGAAAGACACAGAACG |
| CS2-M3 | GGAUGCAGAGAACGAAAGUUCCAUUACGCAUCCAAAUCGACGAAAGACACAGAACG |
| CS2-M4 | GGAUGCAGAGAACGAAAGUUCCAUGCCGCAUCCAAAUCGACGAAAGACACAGAACG |

Docking sequence used for immobilization is underlined. Mutated bases are highlighted in yellow.

**Table S2.** Oligo DNAs used for library-vs-library in vitro selection

| DNA | DNA sequence (5’-3’) |
| --- | --- |
| EcoRI-T7PD_Fw | GATCCGAATTCTGGTGGCGGTGGTTCT |
| L7Ae-Fr1_Rv | TGGTACCTTTCTTGACCTTACCGCTC |
| L7Ae-Fr2_Fw | CGCGGGTTGGCAAAGCTGGTATATA |
| L7Ae-Fr2_Rv | GCCCACGGCTCGGCCCAGAT |
| L7Ae-Fr3_Fw | GCGTCAGCCGCTATAATAAATGAAGG |
| HindIII-T7PD_Rv | ACCCAAGCTTATTAAGCGCCTTTCTCAAAT |
| L7Ae-Fr2_lib_Fw | AGGTCAAGAAAGGTACCAATNNKACTACTNNKGCGGTGNNKCGCGGGTTGGCAAAGCTGG |
| L7Ae-Fr3_lib_Fw | ATCTGGGCCGAGCCGTGGGCNNKNNKNNKNNKNNKGCGTCAGCCGCTATAATAAA |
| N20L_Fw | aatttaatacgactcactataGGCAGAGAAAGGCCATACAATCATTGG |
| N20L_lib_Rv | CGTTCTGTGTCTTTCGTCGATGCCACTGGNNNNNNNNNNNNNNNNNNNNCCAATGATTGTATGGCCTTTCTCTGCC |
| N20L_Rv | CGTTCTGTGTCTTTCGTCGATGCCACTGG |
| N20L_MiSeq_bc_RT | ACACGACGCTCTTCCGATCT-[barcode]-CGTTCTGTGTCTTTCGTCGATG |
| N20L_MiSeq_P7_F | CAAGCAGAAGACGGCATACGAGATAGAGAGACTAGGCAGAGAAAGGCCATACAA |
| MiSeq_R1seq_P5_R | AATGATACGGCGACCACCGAGATCTACACACACTCTTTCCCTACACGACGCTCTTCCGATC |
| R1_bc_LS_Fw | TACACGACGCTCTTCCGATCT-[barcode]-TAAGGTCAAGAAAGGTACCAAT |
| R2_LS_Rv | GTGACTGGAGTTCAGACGTGTGCTCTTCCGATCTATTTATTATAGCGGCTGACGC |
| P5_R1_Fw | AATGATACGGCGACCACCGAGATCTACACTCTTTCCCTACACGACGCTCTTCCGATCT |
| P7_R2_Rv | CAAGCAGAAGACGGCATACGAGATGTGACTGGAGTTCAGACGTGTG |

**Table S3.** Detailed summary of PD-SELEX parameters

| **Round** | **Negative**  **selection** | **PD pool**  **(PFU)** | **RNA pool**  **(pmol)** | **Binding^*1^** | **Washing^*1^** |
| --- | --- | --- | --- | --- | --- |
| 1 | Mock beads,  Ctrl phage, tRNA | 1.0 × 10^11^ | 500 | 30 min at RT | 0.5 mL × 1 |
| 2 | Mock beads,  Ctrl phage, tRNA | 5.0 × 10^10^ | 200 | 30 min at RT | 0.5 mL × 3 |
| 3 | tRNA | 4.0 × 10^10^ | 100 | 15 min at RT | 0.5 mL × 4 |
| 4 | tRNA | 3.0 × 10^10^ | 50 | 15 min at 37 °C | 0.5 mL × 5 |
| 5 | tRNA | 2.0 × 10^10^ | 25 | 10 min at 37 °C | 0.5 mL × 5 |
| 6 | tRNA | 5.0 × 10^9^ | 10 | 10 min at 37 °C | 0.5 mL × 8 |

^*1^ RT: room temperature. The selection buffer was supplemented with 0.5 M urea in rounds 5 and 6.

**Table S4 (related to Figure 6 and S10).** RNA-RBP binding properties determined by SPR

| **RNA** | **Protein** | ***k*_on_ (M^-1^ s^-1^)** | ***k*_off_ (s^-1^)** | ***K*_D_ (M)** |
| --- | --- | --- | --- | --- |
| CS1 | LS4^†^  LS12  L7Ae | (2.00 ± 0.07) × 10^7^  -  (1.23 ± 0.72) × 10^6^ | (1.36 ± 0.15) × 10^-4^  -  (1.71 ± 0.71) × 10^-3^ | (6.82 ± 0.98) × 10^-12^  (2.75 ± 0.12) × 10^-8 ‡^  (1.49 ± 0.31) × 10^-9^ |
| CS1-M1 | LS4  LS12  L7Ae | (1.85 ± 0.26) × 10^6^  -  (3.04 ± 0.11) × 10^5^ | (3.08 ± 0.69) × 10^-4^  -  (5.48 ± 1.28) × 10^-4^ | (1.71 ± 0.61) × 10^-10^  (7.70 ± 2.24) × 10^-9 ‡^  (1.81 ± 0.49) × 10^-9^ |
| CS1-M2 | LS4  LS12  L7Ae | (3.43 ± 0.98) × 10^5^  N.D.  N.B. | (7.42 ± 1.36) × 10^-4^  -  - | (2.21 ± 0.27) × 10^-9^  -  - |
| CS1-M3 | LS4  LS12  L7Ae | N.B.  N.B.  N.B. | -  -  - | -  -  - |
| CS1-M4 | LS4  LS12  L7Ae | N.D.  -  N.D. | -  -  - | -  (2.55 ± 0.97) × 10^-7 ‡^  - |
| CS1-M2a | LS4  LS12  L7Ae | (1.91 ± 0.43) × 10^6^  -  (2.94 ± 0.92) × 10^5^ | (4.53 ± 1.04) × 10^-4^  -  (3.34 ± 0.57) × 10^-3^ | (2.43 ± 0.60) × 10^-10^  (7.30 ± 3.76) × 10^-9 ‡^  (1.26 ± 0.65) × 10^-8^ |
| CS1-M2b | LS4  LS12  L7Ae | (1.77 ± 1.11) × 10^6^  -  (8.42 ± 2.52) × 10^4^ | (1.37 ± 0.45) × 10^-4^  -  (1.80 ± 0.06) × 10^-3^ | (8.65 ± 2.86) × 10^-11^  (1.10 ± 0.30) × 10^-7 ‡^  (2.22 ± 0.59) × 10^-9^ |
| CS1-M2c | LS4  LS12  L7Ae | (3.82 ± 0.27) × 10^5^  -  (5.41 ± 1.48) × 10^4^ | (2.79 ± 0.45) × 10^-4^  -  (2.21 ± 0.80) × 10^-3^ | (7.29 ± 0.67) × 10^-10^  (4.97 ± 1.95) × 10^-8 ‡^  (4.46 ± 2.71) × 10^-8^ |
| CS1-M2d | LS4  LS12  L7Ae | (2.89 ± 0.52) × 10^5^  -  (2.06 ± 0.24) × 10^4^ | (2.49 ± 0.15) × 10^-3^  -  (4.64 ± 0.72) × 10^-3^ | (8.85 ± 1.97) × 10^-9^  (3.82 ± 0.71) × 10^-7 ‡^  (2.29 ± 0.62) × 10^-7^ |
| CS1-M2e | LS4  LS12  L7Ae | (3.88 ± 0.23) × 10^5^  -  (4.81 ± 0.51) × 10^4^ | (2.61 ± 0.20) × 10^-4^  -  (6.68 ± 2.21) × 10^-3^ | (6.73 ± 0.10) × 10^-10^  (1.66 ± 0.46) × 10^-7 ‡^  (1.42 ± 0.61) × 10^-7^ |
| CS2 | LS4  LS12^†^  L7Ae | -  (4.96 ± 3.48) × 10^7^  (1.69 ± 1.08) × 10^7^ | -  (3.26 ± 1.58) × 10^-4^  (8.25 ± 1.84) × 10^-5^ | (1.14 ± 0.13) × 10^-7 ‡^  (7.24 ± 2.29) × 10^-12^  (5.71 ± 2.56) × 10^-12^ |
| CS2-M1 | LS4  LS12  L7Ae | -  (7.15 ± 1.28) × 10^6^  (1.01 ± 0.88) × 10^6^ | -  (1.12 ± 0.12) × 10^-3^  (1.89 ± 0.53) × 10^-4^ | (9.58 ± 0.92) × 10^-7 ‡^  (1.61 ± 0.40) × 10^-10^  (1.25 ± 0.45) × 10^-10^ |
| CS2-M2 | LS4  LS12  L7Ae | N.B.  N.B.  N.B. | -  -  - | -  -  - |
| CS2-M3 | LS4  LS12  L7Ae | N.D.  (1.03 ± 0.13) × 10^6^  (1.29 ± 0.07) × 10^5^ | -  (6.14 ± 0.24) × 10^-3^  (2.58 ± 0.09) × 10^-2^ | -  (6.02 ± 0.54) × 10^-9^  (2.00 ± 0.03) × 10^-7^ |
| CS2-M4 | LS4  LS12  L7Ae | N.B.  (7.88 ± 1.16) × 10^5^  (1.22 ± 0.19) × 10^5^ | -  (2.17 ± 0.06) × 10^-3^  (6.92 ± 0.97) × 10^-4^ | -  (2.79 ± 0.32) × 10^-9^  (5.69 ± 0.55) × 10^-9^ |
| Box C/D Kt | LS4  LS12  L7Ae^†^ | -  -  (1.41 ± 1.11) × 10^7^ | -  -  (2.77 ± 1.75) × 10^-4^ | (5.99 ± 1.64) × 10^-7 ‡^  (1.45 ± 0.07) × 10^-8 ‡^  (2.13 ± 0.43) × 10^-11^ |

*k*_on_, *k*_off_, *K*_D_ values are means and standard deviations of at least two independent experiments. No binding (N.B.) observed at the protein concentrations tested. Weak binding observed but *K*_D_ value could not be determined (N.D.). ^†^ Identical to data in the main text (Table 1 and 2). ^‡^ equilibrium analysis.

**SUPPLEMENTARY REFERENCES**

1. Jarmoskaite, I., AlSadhan, I., Vaidyanathan, P.P. and Herschlag, D. (2020) How to measure and evaluate binding affinities. *Elife*, **9**, e57264.

2. Schneider, C.A., Rasband, W.S. and Eliceiri, K.W. (2012) NIH Image to ImageJ: 25 years of image analysis. *Nat. Methods*, **9**, 671-675.

3. Gasteiger, E., Hoogland, C., Gattiker, A., Duvaud, S., Wilkins, M.R., Appel, R.D. and Bairoch, A. (2005) Protein Identification and Analysis Tools on the ExPASy Server. *The Proteomics Protocols Handbook*, 571-607.

4. Shi, Y., Mowery, R.A., Ashley, J., Hentz, M., Ramirez, A.J., Bilgicer, B., Slunt-Brown, H., Borchelt, D.R. and Shaw, B.F. (2012) Abnormal SDS-PAGE migration of cytosolic proteins can identify domains and mechanisms that control surfactant binding. *Protein Sci.*, **21**, 1197-1209.

5. Condron, B.G., Atkins, J.F. and Gesteland, R.F. (1991) Frameshifting in gene 10 of bacteriophage T7. *J. Bacteriol.*, **173**, 6998-7003.

6. UniProt, C. (2021) UniProt: the universal protein knowledgebase in 2021. *Nucleic Acids Res.*, **49**, D480-D489.

7. Larkin, M.A., Blackshields, G., Brown, N.P., Chenna, R., McGettigan, P.A., McWilliam, H., Valentin, F., Wallace, I.M., Wilm, A., Lopez, R. *et al.* (2007) Clustal W and Clustal X version 2.0. *Bioinformatics*, **23**, 2947-2948.
